# Prediction of chronological and biological age from laboratory data

**DOI:** 10.18632/aging.102900

**Published:** 2020-05-05

**Authors:** Luke Sagers, Luke Melas-Kyriazi, Chirag J Patel, Arjun K Manrai

**Affiliations:** 1 Computational Health Informatics Program, Boston Children’s Hospital, Boston, MA 02215, USA; 2 Department of Biomedical Informatics, Harvard Medical School, Boston, MA 02115, USA; 3 Department of Pediatrics, Harvard Medical School, Boston, MA 02115, USA; 4 Department of Mathematics, Harvard University, Cambridge, MA 02138, USA

**Keywords:** biomarkers, machine learning, computational models, diversity, big data

## Abstract

Aging has pronounced effects on blood laboratory biomarkers used in the clinic. Prior studies have largely investigated one biomarker or population at a time, limiting a comprehensive view of biomarker variation and aging across different populations. Here we develop a supervised machine learning approach to study aging using 356 blood biomarkers measured in 67,563 individuals across diverse populations. Our model predicts age with a mean absolute error (MAE), or average magnitude of prediction errors, in held-out data of 4.76 years and an R^2^ value of 0.92. Age prediction was highly accurate for the pediatric cohort (MAE = 0.87, R^2^ = 0.94) but inaccurate for ages 65+ (MAE = 4.30, R^2^ = 0.25). Variability was observed in which biomarkers carry predictive power across age groups, genders, and race/ethnicity groups, and novel candidate biomarkers of aging were identified for specific age ranges (e.g. Vitamin E, ages 18-44). We show that predictors for one age group may fail to generalize to other groups and investigate non-linearity in biomarkers near adulthood. As populations worldwide undergo major demographic changes, it is increasingly important to catalogue biomarker variation across age groups and discover new biomarkers to distinguish chronological and biological aging.

## INTRODUCTION

Aging has pronounced effects on blood laboratory biomarkers used in the clinic such as testosterone [1] and plasma fibrinogen [2]. As worldwide populations undergo major demographic and aging shifts [3], it will be increasingly important to understand how aging relates to not just single blood biomarkers but combinations of many blood biomarkers together, particularly for age-associated diseases that lack inexpensive and noninvasive tools for early detection and staging such as Alzheimer’s disease [4]. Studies of laboratory analytes and aging have traditionally considered a single analyte at a time [5–7] and have been limited in their inclusion of demographically diverse groups [8]. Simultaneously modeling many blood biomarkers together across population groups paints a more complete picture of health and disease and enables the systematic study of differences resulting from the definitions of age based on time since birth (“chronological age”) and as a cumulative measure of biological wear and tear (“biological age”) [9].

Recently, machine learning and statistical methods have enabled agnostic, data-driven approaches to age prediction based on methylation [10, 11], transcriptomic [12], and retinal imaging data [13]. For example, in 2018, researchers at Google used deep neural networks to analyze retinal fundus images to predict cardiovascular risk factors including a patient’s age [13]. While machine learning has been widely applied to fields such as medical imaging [14, 15] and speech recognition [16, 17], it is comparatively underapplied in the study of blood laboratory biomarkers [18, 19], which may be among the cheapest to measure in individuals.

In this study, we apply supervised machine learning methods to 356 blood laboratory measures from 67,563 individuals. Our aim is to systematically study the predictive capacity of individual and large collections of blood laboratory biomarkers for predicting chronological age across the lifespan. We compute aging curves for all blood laboratory measures and assess whether changes in the predictive power of individual biomarkers are consistent across different populations with respect to gender, race, and income. We document how age predictors that perform highly accurately in one population may generalize poorly to different populations and use piecewise linear regression methods to investigate significant age-related changes in the trajectories of laboratory analytes. Our results identify clear demographic structure embedded in blood laboratory data and show that we are able to predict chronological age from laboratory analytes with high accuracy, which compares favorably to top predictors in the field [20].

## RESULTS

### Age is highly predictable from blood laboratory analytes

**Figure 1:**
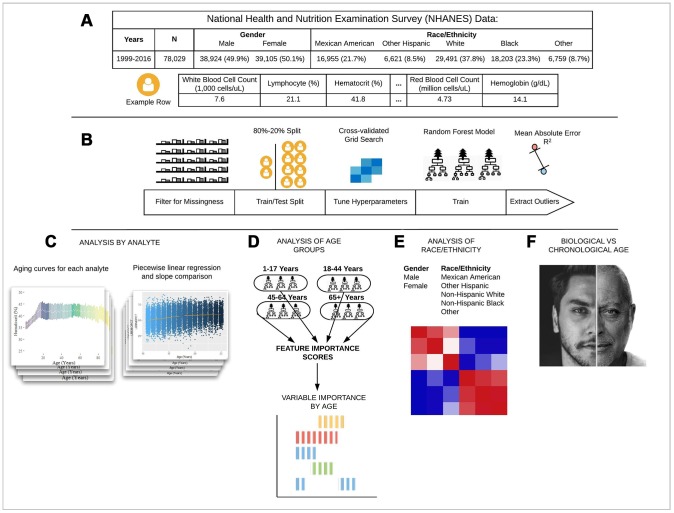
**Schematic overview of our study.** (**A**) The CDC NHANES datasets from 1999-2016 (N refers to size before filtering) were used in our analyses; shown are summary statistics and an example row for a single individual in the dataset. (**B**) An overview of the machine learning pipeline used in the study. We filtered on a set missingness criteria (Methods) and then separated individuals into an 80/20 train/test split. We used a random forest model with hyperparameters tuned using a cross-validated grid search. After training the model we tested using cross-validation and the 20% held-out test set and analyzed outliers. (**C**) Aging curves for individual analytes were computed and analyzed for linear and non-linear trends. Piecewise regression analysis and breakpoint estimation were used to estimate breakpoints and compare slopes separated by breakpoints. (**D**) Models were trained separately for four U.S. Census age groups and feature importance scores were computed for each age group. (**E**) Models were trained on subgroups of the dataset separated by race and gender. The feature importance scores were calculated for each model and compared across race/gender groups. (**F**) Analyses of the trajectories of analytes across age ranges were used to compare chronological vs. biological definitions of age.

We trained a random forest model [21] to predict chronological age (in years) using data from 67,563 individuals ranging in age from 1 to 85 years (mean: 36.2, standard deviation: 23.1) from nine CDC National Health and Nutrition Examination Survey (NHANES) cohorts spanning 1999-2016 (Figure 1), a representative sample of the non-institutionalized population of the United States. The model included 356 features consisting of laboratory analytes (e.g. serum glucose, creatinine). Many of the analytes contained a large proportion of missing data (Supplementary Table 3), which was dealt with by imputing missing values using mean imputation. We evaluated model performance both using five-fold cross-validation and held-out data (Methods). Hyperparameters were selected by grid search (Methods). We define our baseline model for chronological age prediction as a linear regression model without regularization, using age as the response variable and the 356 laboratory analytes as covariates. Mean absolute error (MAE) for the baseline linear regression model was 10.53 (SE: 0.07) years in five-fold cross-validation and 10.52 years in the 20% held-out dataset. The R^2^ for the baseline model was 0.63 (SE: 0.01) in the five-fold cross-validation and 0.62 in the held-out set. In our best random forest model, MAE was 4.80 (SE: 0.013) years in cross-validation and 4.76 years in the 20% held- out dataset. The R^2^ from the random forest model was 0.92 (SE: 0.0005) in the five-fold cross-validation and 0.92 in the held-out set.

**Figure 2:**
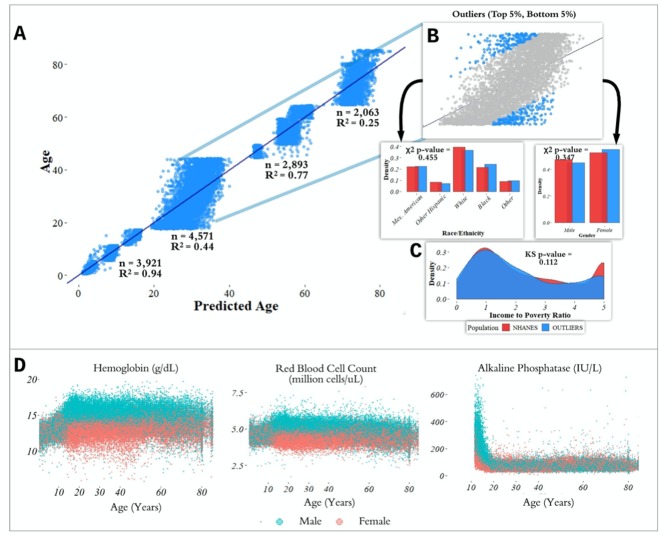
**Performance of prediction model across age groups.** (**A**) Actual age vs. predicted age from the random forest model with R2 and sample size (n) for each age range in the test set. (**B**) Observations with a residual error falling in the top 5% or bottom 5% were identified and compared to the overall NHANES population. (**C**) Gender, race, and income to poverty ratio distributions were compared between outliers and the overall NHANES population. (**D**) Analyte levels by age, colored by gender. Hemoglobin, red blood cell count, and alkaline phosphatase were selected to represent contrasting patterns in the separability of males and females at different age ranges.

We also trained separate random forest models for the four main United States Census [22] age groups: [1,18), [18,45), [45,65), 65+. The predictive accuracy of the models differed substantially across age groups (pairwise R^2^ comparisons were significant while adjusting for multiple comparisons; Methods) (Figure 2; Table 1). The model for the [1,18) age group had the Random forest models were trained on data from individuals across different age ranges within the dataset. Mean absolute error and R-squared are shown in the table as measurements of model performance on held-out data.

**Table 1:** Performance of random forest models trained on different age ranges.

**RF Models Trained on Different Age Ranges**		
**Age Range**	**Mean Absolute Error**	**R^2^**
1-17 years	0.87	0.94
18-44 years	5.15	0.44
45-64 years	2.20	0.77
65+ years	4.30	0.25
1-85 years (Overall)	4.76	0.92

Random forest models were trained on data from individuals across different age ranges within the dataset. Mean absolute error and R-squared are shown in the table as measurements of model performance on held-out data.

most accurate predictions of the four and the model trained on the 65+ age group had the least accurate predictions of the four, as measured by R^2^ for age prediction in years. In the held-out dataset, the MAE for [1,18) was 0.87 years and the R^2^ was 0.94. For [18,45), the MAE in the held-out dataset was 5.15 years and the R^2^ value was 0.44; for [45,65), the MAE was 2.20 years and R^2^ value was 0.77; for the 65+ cohort, the MAE was 4.30 years and the R^2^ value was 0.25 (Figure 2).

### Feature importance differs substantially across age groups

In order to estimate the predictive power of specific laboratory analytes in a comparable manner, we computed variable importance scores (calculated using the decrease in node impurity using the Gini impurity measure; Methods) for each of the age-specific models across the 356 laboratory analytes. We define the Top-10 set for each age bin as the 10 laboratory analytes with largest variable importance scores for the random forest model trained on that age group, denoted e.g. Top-10_[1,18)_ for the [1,18) age group (similarly for Top-5). We computed relative variable importance scores for each analyte for each age range, and the total relative importance of the Top-5 and Top-10 sets for each age group, denoted e.g. |Top-10_[1,18)_| for the [1,18) age range.

**Figure 3:**
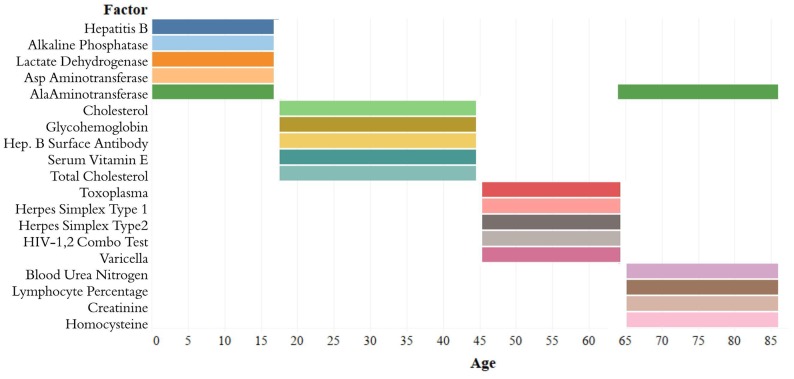
**Top-5 variables (based on feature importance score) across age bins.** The Top-5 variables based on feature importance scores across the four age groups ([1,18), [18,45), [45,65), 65+) are shown. The variables are different for each age group except for alanine aminotransferase, which is present in the top variables of both [1,18) and 65+ age groups.

The analytes in the Top-5 differed substantially across age ranges (Figure 3). The only analyte that appears in the Top-5 for multiple age groups is alanine aminotransferase, which appears in both the [1,18) and 65+ groups. |Top-10_[1,18)_| was 0.751, |Top-10_[18,45)_| was 0.294; |Top-10_[45,65)_| was 0.542; |Top-10_65+_| was 0.225. |Top-5_[1,18)_| was 0.567; |Top-5_[18,45)_| was 0.169; |Top-5_[45,65)_| was 0.464; |Top-5_65+_| was 0.144 (Supplementary Table 1). Top-5_[1,18)_ consisted of hepatitis B, alkaline phosphatase, lactate dehydrogenase, aspartate aminotransferase, and alanine aminotransferase; Top-5_[18,45)_ consisted of total cholesterol, serum vitamin E, serum cholesterol, glycohemoglobin, and hepatitis B antibody; Top-5_[45,65)_ consisted of herpes simplex 1, herpes simplex 2, toxoplasma, HIV 1,2 combo test, and varicella. Top-5_65+_ consisted of alanine aminotransferase, blood urea nitrogen, lymphocyte percentage, creatinine, and homocysteine (Figure 3).

### Chronological vs. biological age in laboratory data

In the held-out datasets (total n = 13,513), we defined ‘outliers’ as individuals with residual errors in the top 5% or bottom 5% of their age group, representing approximately 37.1 million individuals in the US (based on NHANES sample weights). For the bottom 5% of each age group, the model underestimated their age by an average of 2.20 years (sd = 0.50) for [1,18); 10.9 years (sd = 1.64) for [18,45); 6.38 years (sd = 1.80) for [45,65); and 8.64 years (sd = 1.09) for 65+. For the top 5% of each age group, the model overestimated the age of outliers by an average of 2.47 years (sd = 0.89) for [1,18); 12.23 years (sd = 1.91) for [18,45); 5.52 years (sd = 0.72) for [45,65); and 9.07 years (sd = 1.04) for 65+. We compared the outliers from each age bin with the remaining individuals in the held-out dataset to assess differences in demographic features between these groups (Figure 2). After correcting for multiple comparisons, we found no significant differences between the outlier populations from each age bin and the rest of the individuals from that age bin in gender, race, and income to poverty ratio distributions.

In order to investigate aging in males and females, we stratified 356 analytes individually by age and gender (Figure 2D, Supplementary Figure 2). We found that several analytes, including major blood labs such as red blood cell count, hemoglobin, and hematocrit, and other labs such as alkaline phosphatase, lactate dehydrogenase, and calcium, showed age-related changes that differed starkly between males and females. For hematocrit, hemoglobin, and red blood cell count, male and female values are homogeneously mixed in younger children, separate in the teenage years until male and female values exhibit different ranges, and then cross over again in the senior years. For alkaline phosphatase, this trend is reversed, and sexual dimorphism is apparent in children below 15 years old, and then gradually reduces with age.

### Feature scores are highly correlated for males and females of different race/ethnicity groups

**Figure 4:**
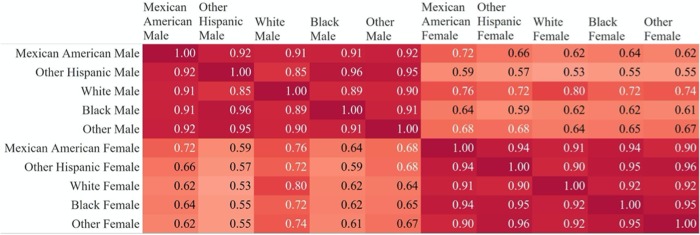
**Correlations of feature importance scores across gender and race subgroups.** Pairwise correlations between feature importance scores from random forest models trained on subsets of the data (separated by gender and race/ethnicity). Correlations are consistently stronger across race groups for the same gender.

We trained separate chronological age predictors for different subpopulations spanning combinations of gender (male, female) and race (Mexican American, Other Hispanic, Non-Hispanic White, Non-Hispanic Black, Other) groups in the NHANES population. We computed correlation coefficients to compare the variable importance scores for each pair of subpopulations (e.g. Mexican American Females and Black Males). Figure 4 shows all pairwise correlations between the feature importance scores across the 10 groups. Pairs of race/ethnicity groups of the same gender all had correlation coefficients above 0.85, with the majority above 0.9. Correlations between feature importance scores of males and females in the same race groups and across race groups were considerably lower, ranging from 0.53-0.80. The strongest correlation between male and female feature importance scores across race groups occurred between white males and white females (0.80).

### Models accurate for one age group fail to generalize to other age groups

We tested whether Top-5 and Top-10 for each age group could predict chronological age accurately for other age groups. Table 2 contains the MAE values for models containing only the Top-5 and Top-10 variables when trained and tested on other age groups. The best performing Top-5 model for each age group was the model trained using the Top-5 from that age group. The same was true for the Top-10 models. For the [1,18) age group, the best Top-5 model gave a MAE of 1.35 and the best Top-10 model gave a MAE of 1.16. For the [18,45) age group, the best Top-5 model gave a MAE of 6.41 and the best Top-10 model gave a MAE of 5.51. For the [45,65) age group, the best Top-5 model gave a MAE of 3.28 and the best Top-10 model gave a MAE of 2.91. And for the 65+ age group, the best Top-5 model gave a MAE of 4.63 and the best Top-10 model gave a MAE of 4.49. These results demonstrate that an analyte’s predictive power is not necessarily the same for different age bins and that the set of most predictive analytes is not consistent across age bins.

**Table 2:** Mean absolute errors (MAEs) for models trained and applied on different age ranges.

**MAE by Age Bin**				
**Age Group Tested**				
**Model Trained On**	**[1-18) Years**	**[18-45) Years**	**[45-65) Years**	**65+ Years**
**Top-5_[1,18)_**	**1.35**	7.17	5.11	4.88
**Top-5_[18,45)_**	1.60	**6.41**	5.12	5.26
**Top-5_[45, 65)_**	2.21	7.18	**3.28**	NA
**Top-5_65+_**	1.94	7.02	4.96	**4.63**
**Top-10_[1,18)_**	**1.16**	6.36	4.79	4.61
**Top-10_[18,45)_**	1.33	**5.51**	3.19	5.00
**Top-10_[45, 65)_**	2.12	6.80	**2.91**	NA
**Top-10_65+_**	1.53	6.57	4.79	**4.49**

Models containing only the Top-5 and Top-10 variables were trained in one age group and tested on all age groups. MAEs are from testing on the 20% held-out dataset in each case.

### Widespread non-linearity in analyte aging trajectories

**Figure 5:**
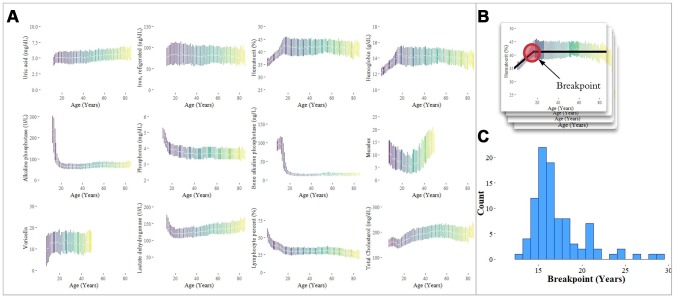
**Analysis of individual analytes for linear and non-linear trends.** (**A**) Laboratory analytes exhibit clear linear and non-linear trends with respect to age. The interquartile ranges of analyte values, plotted by age, are shown for selected analytes. Analytes were selected from our analysis of the Top-10 feature importance scores for each age group, and exhibit linearity, piecewise linearity, power, and U-shaped curves. (**B**) Breakpoints were estimated using piecewise linear regression. (**C**) The distribution of breakpoints for 94 analytes with a significant difference in slope around the breakpoint is shown with a median estimated breakpoint of 16.4 years.

Having observed strong predictive value in the pediatric cohort, we sought to identify significant transitions in biomarker trajectories occurring between the ages of 11 and 30 for comparison against traditional age groupings. To do this, we examined 342 laboratory analytes for piecewise linearity by estimating ‘breakpoints’ in the aging curves (analyte level by age) for ages [11, 30] of each analyte using piecewise regression models [23] (Figure 5). Analytes that did not have data for children younger than 18 years were not included in the analysis. We tested the slopes of the regression lines on either side of the breakpoints for differences. Of the 342 analytes tested, 97 were significant (28.4%) for differences in slope at a Bonferroni-adjusted p-value threshold of 1.46 x 10^-4^ (Methods). The median of the 97 breakpoints was 16.4 years with 50% of the breakpoints falling in the range of 15.0-17.7 years. The mode (rounded in years) was 16 years, and the maximum breakpoint was 28.9 years.

In addition to piecewise linearity for the adolescent to adult transition (11 to 30), several different categories of laboratory analyte “aging curves” were observed across the full lifespan, including the following, in order of increasing complexity: (1) linear (e.g. uric acid, iron); (2) piecewise linear (e.g. hematocrit, hemoglobin); (3) power (e.g. alkaline phosphatase, phosphorus); (4) U-shaped (e.g. measles antibody) (Figure 5).

## DISCUSSION

In this study, we show that chronological age can be predicted highly accurately by applying supervised machine learning methods to blood laboratory data. Our analysis of individual laboratory analytes reveals strong linear and non-linear relationships between age and analyte levels that help explain the changing predictive power of different analytes across a lifetime. We also show that for different demographic groups (separated by age range or by gender and race) the set of laboratory analytes with the most power for predicting chronological age varies. With the graying of worldwide populations [3, 24, 25], efforts to understand the aging process in the elderly, especially for age-related diseases like Alzheimer’s and dementia that lack robust biomarkers for early detection, must be accelerated. Our findings around gender and race/ethnicity further underscore the importance of gathering large-scale data from diverse and traditionally underrepresented populations worldwide, and support initiatives such as the NIH’s All of Us Research Program, the UK Biobank, and the China Kadoorie Biobank. Specific applications include testing our age prediction models in these cohorts, re-evaluating traditional reference ranges for diverse groups, and identifying biomarkers that are informative of health risk in different populations [26–28].

The models’ most important features across age groups revealed both known and novel analytes associated with aging. For example, levels of lactate dehydrogenase [29] and alkaline phosphatase [30, 31] are known to vary as children age, and total cholesterol levels rise steadily in adults from ages 18-45 [32]. However, several of the most important analytes were novel. For example, for 18-45 year olds, serum vitamin E was among the Top-5 variables despite little evidence to suggest that levels vary significantly within this age range [33, 34]. Vitamin E acts as an antioxidant, enhances lymphocyte proliferation, and inhibits platelet adhesion [35]. Vitamin E would likely not have been among the top analytes identified as relevant in aging when studied individually but was identified by our model when analyzed in conjunction with many other analytes. Future analyses that stratify by disease outcomes may suggest analytes that work in concert with Vitamin E to affect aging.

In the elderly, the top variables identified by our model were largely consistent with prior literature. Significant changes in levels of blood urea nitrogen [36], lymphocyte percentage [37, 38], creatinine [36, 39], homocysteine [40] and alanine aminotransferase [41–43] are associated with the declining function of the liver, kidneys, immune system, and heart that may be expected with aging. Despite these known associations, the model still performed poorly in predicting age for the 65+ age group. Thus, the model was able to identify analytes relevant to aging but unable to use that information to predict a chronological age precisely. This suggests the need to study other blood biomarkers and data types in the elderly population in order to identify more predictive biomarkers of chronological and biological aging and look for biological predictors for age-related diseases like Alzheimer’s and dementia. Further research should also investigate the inconsistencies in biological aging for the elderly relative to their analyte aging trajectories earlier in life and could stratify individuals by their predicted vs. actual age. Ideally, such analyses would include longitudinal and carefully measured outcomes.

We found that the variables that predict age well vary substantially with age. This effect is seen in the differences in the Top-5 most important variables from models trained on different age ranges as well as the piecewise linear regression results. Further research could investigate precisely when these variables gain and lose predictive power across a person’s lifetime. Understanding these ‘biological transitions’, including when they happen, how suddenly they occur, and which analytes are involved may lead to new insights into the milestones of aging and the consequences associated with it.

The model trained on the entire population and the model trained on just the pediatric cohort (ages [1,18)) were substantially more accurate for predicting ages in the range [1,18) than models trained on any other age range. Even with limited data for the youngest children (i.e. many NHANES lab tests are not administered to children below 12 years), we were able to predict age in this pediatric group to within a year (MAE = 0.87). These levels of accuracy reveal a strong relationship between a child’s collection of laboratory analytes and their chronological age and motivate the development of models based on data of higher temporal resolution, younger populations, and combinations of many lab analytes. Our age predictor has the potential to improve understanding of child development, flag aberrant aging patterns in children (which may be associated with other conditions), and help establish clinical ranges of normality for groups of biomarkers, just as head circumference, weight, and height are used in clinical practice to survey a growing child’s health and nutrition.

The varying predictive ability of individual laboratory analytes across demographics and age ranges illustrates that models that can predict age well for one group of people may fare poorly in other groups. This has potential impact on the use (and misuse) of current age prediction approaches (e.g., Putin et al [18]). With a large set of variables, the model space is exponentially large, and brute force methods for finding the best possible model for a specific group among all models become computationally overwhelming. The question of how to appropriately catalogue, parameterize, and search this model space is worth addressing so age prediction can be systematically explored and both optimal and problematic models can be identified.

Limitations of the present study include primarily the substantial amount of missing or incomplete data in the CDC NHANES cohorts, which we addressed with imputation. Such missing or imputed data often encodes the structure of the data collection process in the dataset itself, as opposed to the blood laboratory values in isolation [44]. For NHANES, there are many laboratory analytes that are measured in certain age ranges and not tested in others. These age-specific tests can be used by a model as discriminants for predicting age within different age ranges and thus bias prediction. We performed sensitivity analyses by restricting to analytes and sets of analytes with complete data across age ranges, but there is no substitute for collecting more complete data.

Modeling the relationship between a group of many biological markers and an individual’s chronological age raises questions about the nature of aging. In medicine, standards and reference ranges are often set with respect to a person’s “years since birth”, even though the biological state of two people born on the same day may be quite different. While age and demographic-related changes in blood laboratory biomarkers have been well documented for single analytes, our study reveals that large collections of lab analytes better predict chronological age and exhibit clear non-linear demographic structure. Translating biomarker studies across age groups is likely to require a comprehensive and diverse view of the aging process that considers varying predictability of biomarkers across the lifespan.

## MATERIALS AND METHODS

### Data

We collected blood laboratory analyte measurements and demographic data from nine waves of the Centers for Disease Control and Prevention (CDC) National Health and Nutrition Examination Survey (NHANES) including the following cohorts: 1999-2000, 2001-2002, 2003-2004, 2005-2006, 2007-2008, 2009-2010, 2011-2012, 2013-2014, 2015-2016. Observations with zero or missing two-year survey weights were removed and all variable names with prefix ‘LBX’ were retained (Supplementary Table 2). Laboratory analytes with greater than 95% missingness were removed (i.e. analytes measured in < 3,901 of individuals), and individuals with fewer than 20 measured labs were also removed (Supplementary Table 3 shows the number of missing values for each laboratory variable). These criteria allowed for analytes to be included in the model with a large proportion of missing values (many contained over 50% missing values). We imputed all missing values using mean imputation, where each missing value was replaced by the mean value for that analyte over all individuals. The final dataset included 67,563 individual and 356 laboratory analytes. Code is available for download here: https://github.com/manrai/Age-Prediction.

### Supervised learning for predicting age from laboratory data

We used random forests [21] to train the main age prediction models in this study. Random forests are machine learning models composed of an ensemble of many decision/regression trees. Each of the individual trees in the “forest” is trained on a bootstrap sample of the training data, while the features used for splitting at each node are selected from a random subset of all possible features. Random forests are robust to outliers and perform well on data with linear and non-linear features.

The random forest model used in this analysis was implemented with the scikit-learn library in Python [45]. The data was partitioned randomly using an 80%-20% train-test split, missing values were imputed using mean imputation, and all variables were normalized. We selected hyperparameters using a grid search method in which the maximum number of trees in the random forest and the maximum number of features selected for evaluation at each tree were iteratively evaluated over 50 combinations and scored using five fold cross-validation while taking into account computational time (grid combinations included: max number of trees = 25, 50, 100, 200, 400, 500; max number of features selected = 1, 5, 10, 25, 50, 100; and bootstrap = True, False). We evaluated model accuracy using five-fold cross-validation (scored with the mean absolute error criterion) on the training data and then tested on the 20% held-out dataset. Ordinary least squares linear regression was used for baseline predictions.

### Statistical analysis

Variable importance was calculated using Gini impurity, or Mean Decrease Gini, as implemented in the *feature_importances_* function in scikit-learn. The importance of a variable *X_m_* is calculated as suggested by Breiman [21, 46, 47]:

$${\text{Imp(X}}_{\text{m}})=\frac{1}{N_{T}}\sum_{T}\sum_{t\in T:v(s_{t})=X_{m}}p(t)\Delta i(s_{t},t)$$

where *N_T_* is the number of trees, *v*(*s_t_*) is the variable in split *s_t_*, and *p*(*t*)Δ*i*(*s_t_*, *t*) is the weighted impurity decrease (using Gini impurity) for all nodes *t* where *X_m_* is used [46]. The sum of variable importance scores across all variables is 1:

$$\sum_{m}^{M}\text{Imp(}X_{{}_{m}})=1$$

For each age range, we defined the total relative importance of the Top-5 and Top-10 variables as:

$$\left|{\text{Top}}_{5}\right|=\sum_{k\in \text{Top-5}}\text{Imp(}X_{m}{)}_{k}$$

$$\left|{\text{Top}}_{10}\right|=\sum_{k\in \text{Top-10}}\text{Imp(}X_{m}{)}_{k}$$

Piecewise regression analysis was carried out using the segmented package [48] in R. Piecewise regression models were used to estimate a breakpoint, in this case an age that marks a change in trajectory, for each of the 342 analytes used in the analysis. Breakpoint estimates and corresponding test statistics were computed using the Davies’ test (via the *davies.test* R function), which tests for non-zero differences in the slope parameter of a segmented relationship between the regression lines on either side of the estimated breakpoint [49]. We used a Bonferroni adjustment to set a threshold for statistical significance at p < 0.05/342, correcting for the 342 analytes.

A vector of feature importance scores was computed for models trained on subgroups consisting of gender and race combinations (10 total subgroups). Pearson product-moment correlations were then computed using pairwise complete importance scores for each subgroup against every other. R^2^ values were computed using predictions in the 20% held-out dataset compared. Chi-squared tests were performed in R using the chisq.test function and the Kolmogorov-Smirnov test was performed using the *ks.test* function in R 50.

## Supplementary Material

Supplementary Figures

Supplementary Table 1

Supplementary Table 2

Supplementary Table 3
