# Supplementary material for: Prediction of chronological and biological age from laboratory data
Source: Aging (Albany NY). 2020 May 5;12(9):7626–38. doi: 10.18632/aging.102900 (PMC7244024; doi:10.18632/aging.102900)
Supplement: Supplementary Figures [file aging-12-102900-s004..pdf]

Supplementary Figure 1. Piecewise linear regression plots for 342 laboratory analytes with lines representing linear regression on either side of the breakpoint.

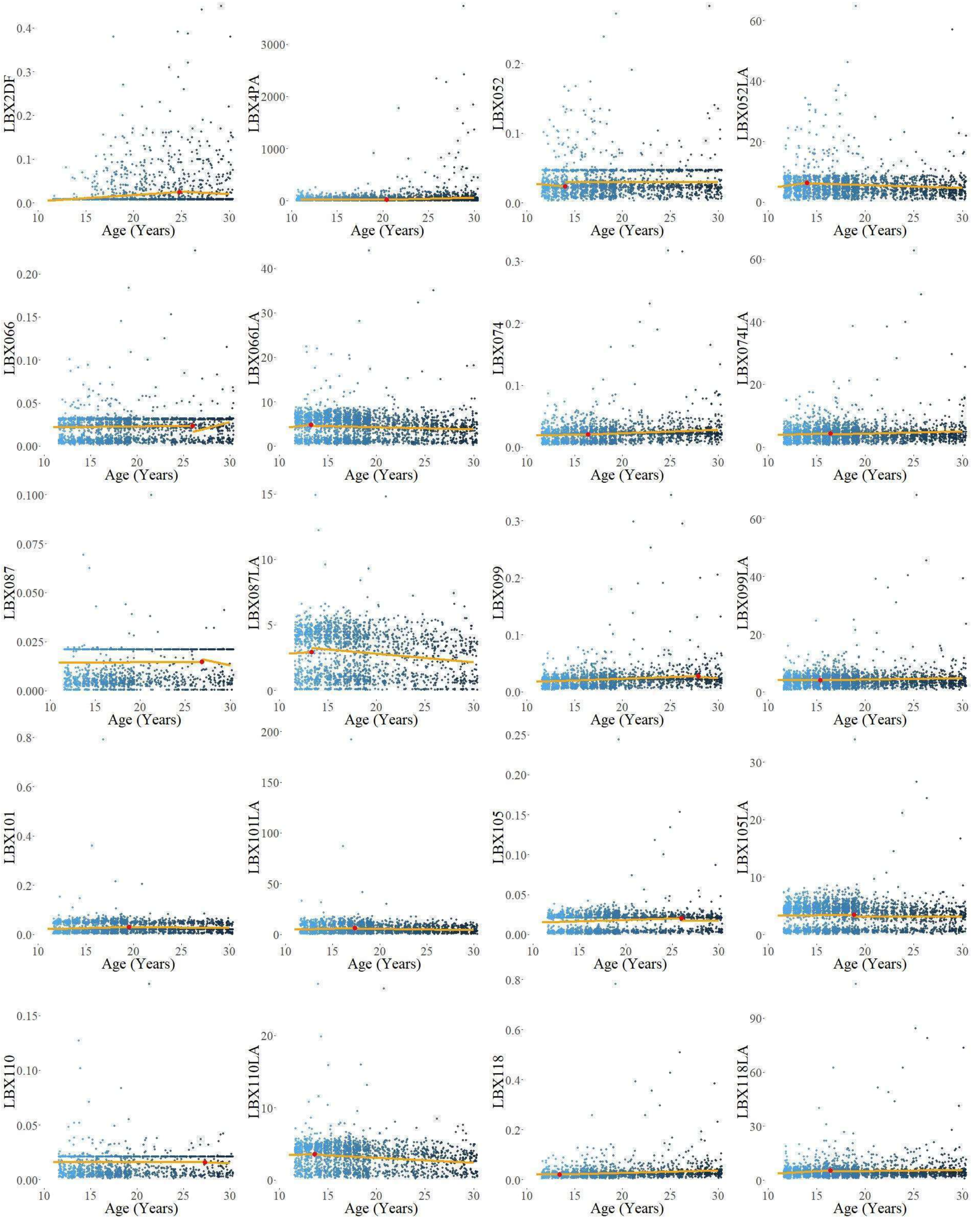
LBX2DF
LBX4PA
LBX052
LBX052LA
LBX066
LBX066LA
LBX074
LBX074LA
LBX087
LBX087LA
LBX099
LBX099LA
LBX101
LBX101LA
LBX105
LBX105LA
LBX110
LBX110LA
LBX118
LBX118LA
Age (Years)

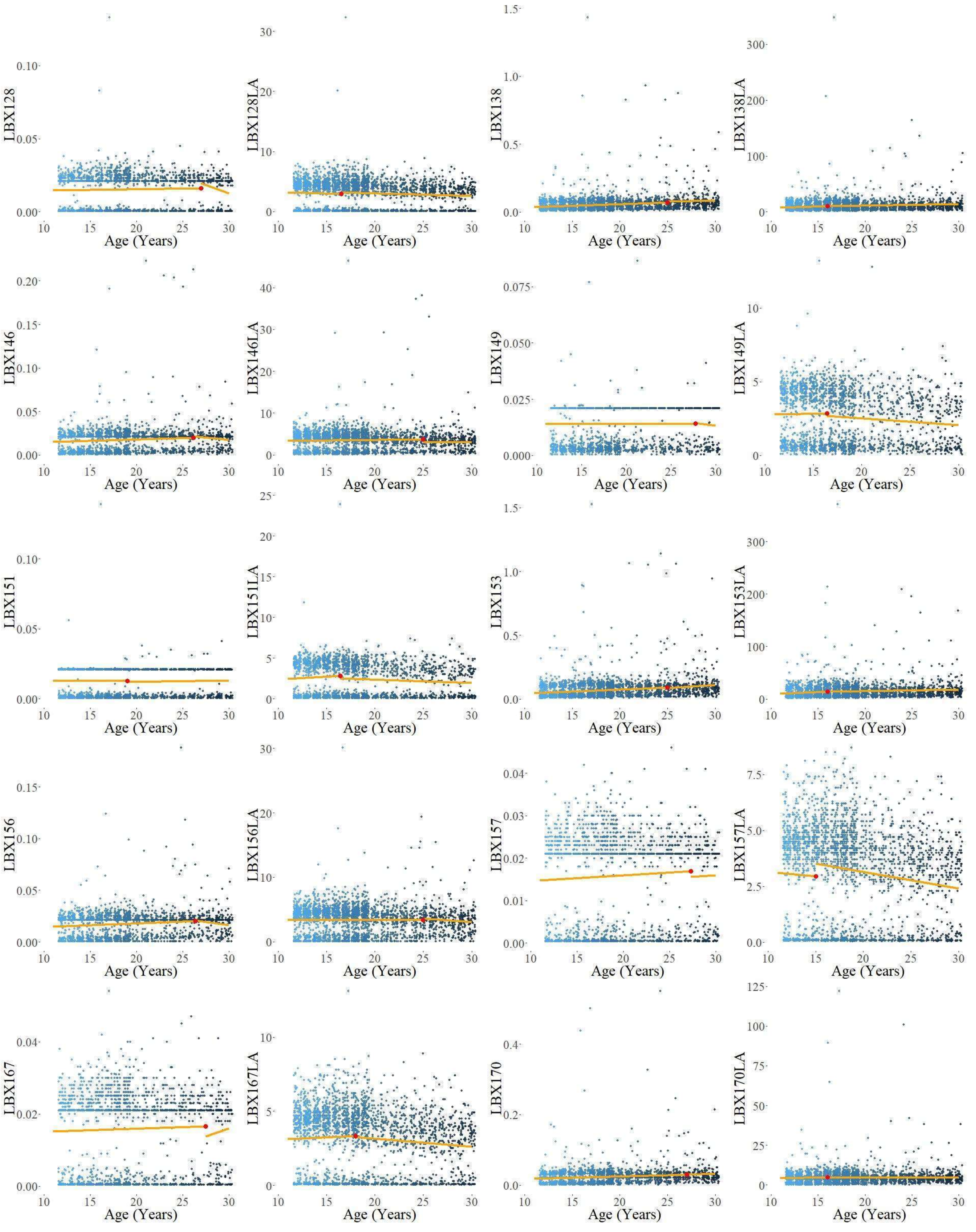

LBX128
LBX128LA
LBX138
LBX138LA
LBX146
LBX146LA
LBX149
LBX149LA
LBX151
LBX151LA
LBX153
LBX153LA
LBX156
LBX156LA
LBX157
LBX157LA
LBX167
LBX167LA
LBX170
LBX170LA
Age (Years)

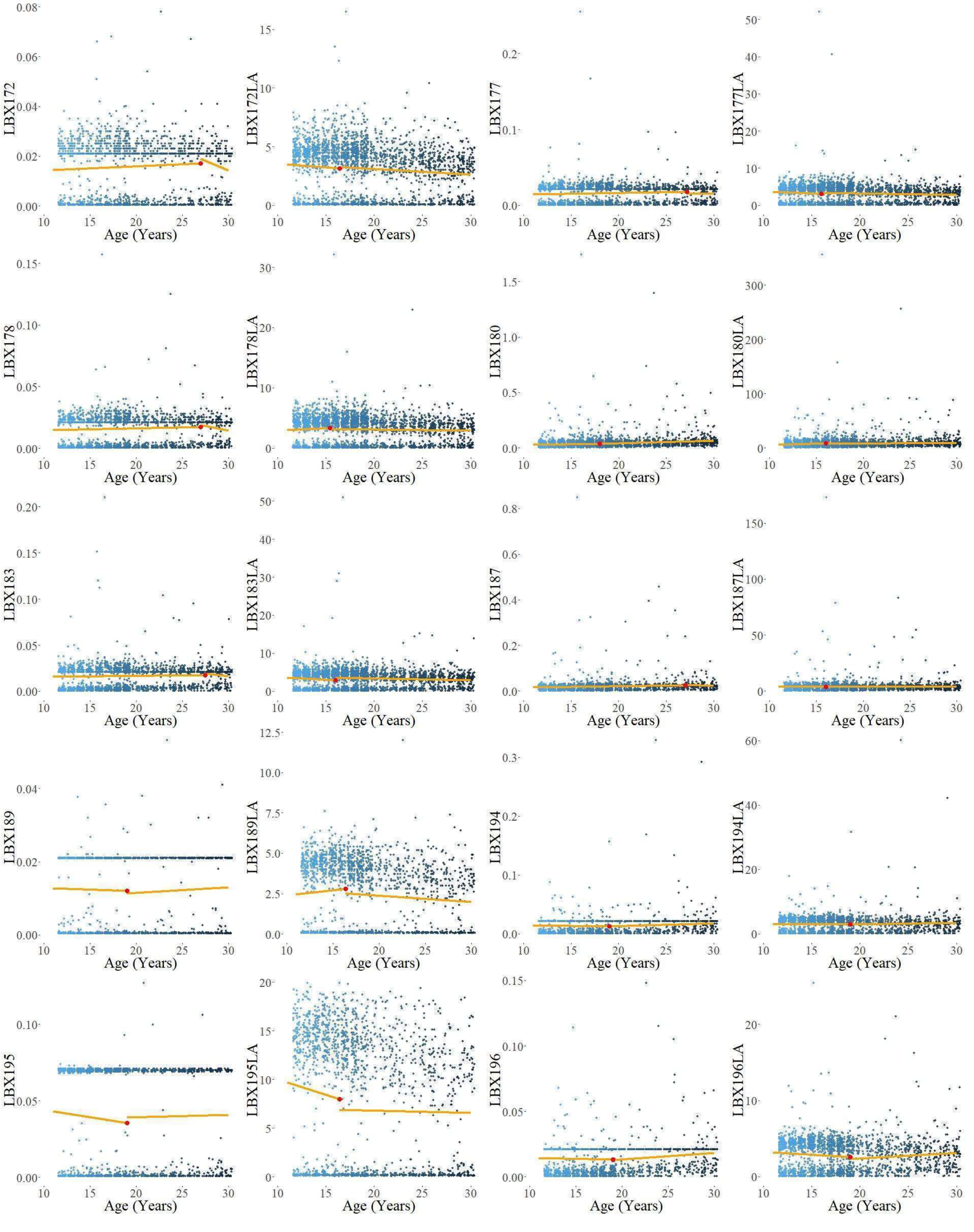
LBX172
LBX172LA
LBX177
LBX177LA
LBX178
LBX178LA
LBX180
LBX180LA
LBX183
LBX183LA
LBX187
LBX187LA
LBX189
LBX189LA
LBX194
LBX194LA
LBX195
LBX195LA
LBX196
LBX196LA
Age (Years)

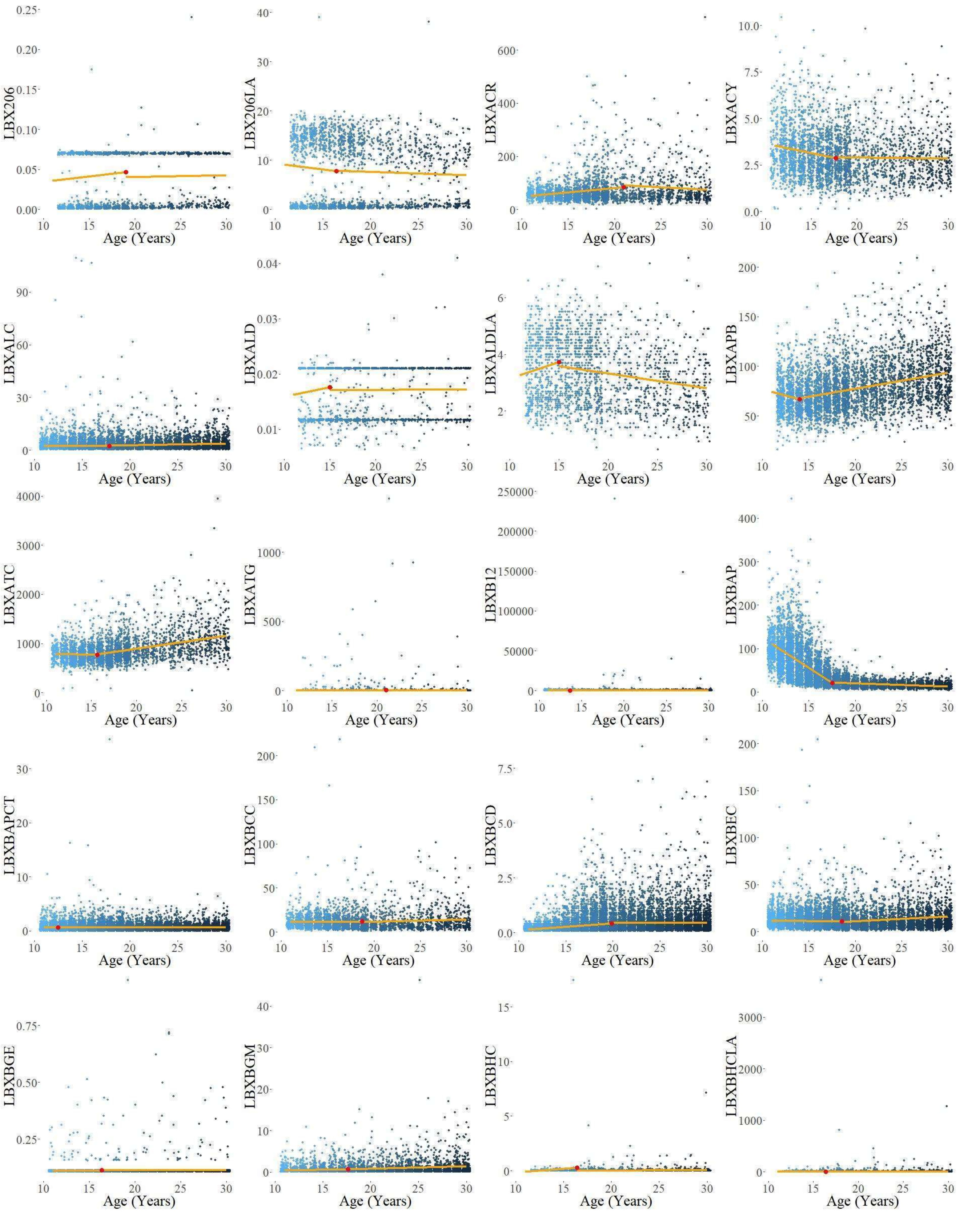
LBX206
Age (Years)
LBX206LA
Age (Years)
LBXACR
Age (Years)
LBXACY
Age (Years)
LBXALC
Age (Years)
LBXALD
Age (Years)
LBXALDLA
Age (Years)
LBXAPB
Age (Years)
LBXATC
Age (Years)
LBXATG
Age (Years)
LBXB12
Age (Years)
LBXBAP
Age (Years)
LBXBAPCT
Age (Years)
LBXBCC
Age (Years)
LBXBCD
Age (Years)
LBXBEC
Age (Years)
LBXBGE
Age (Years)
LBXBGM
Age (Years)
LBXBHC
Age (Years)
LBXBHCLA
Age (Years)

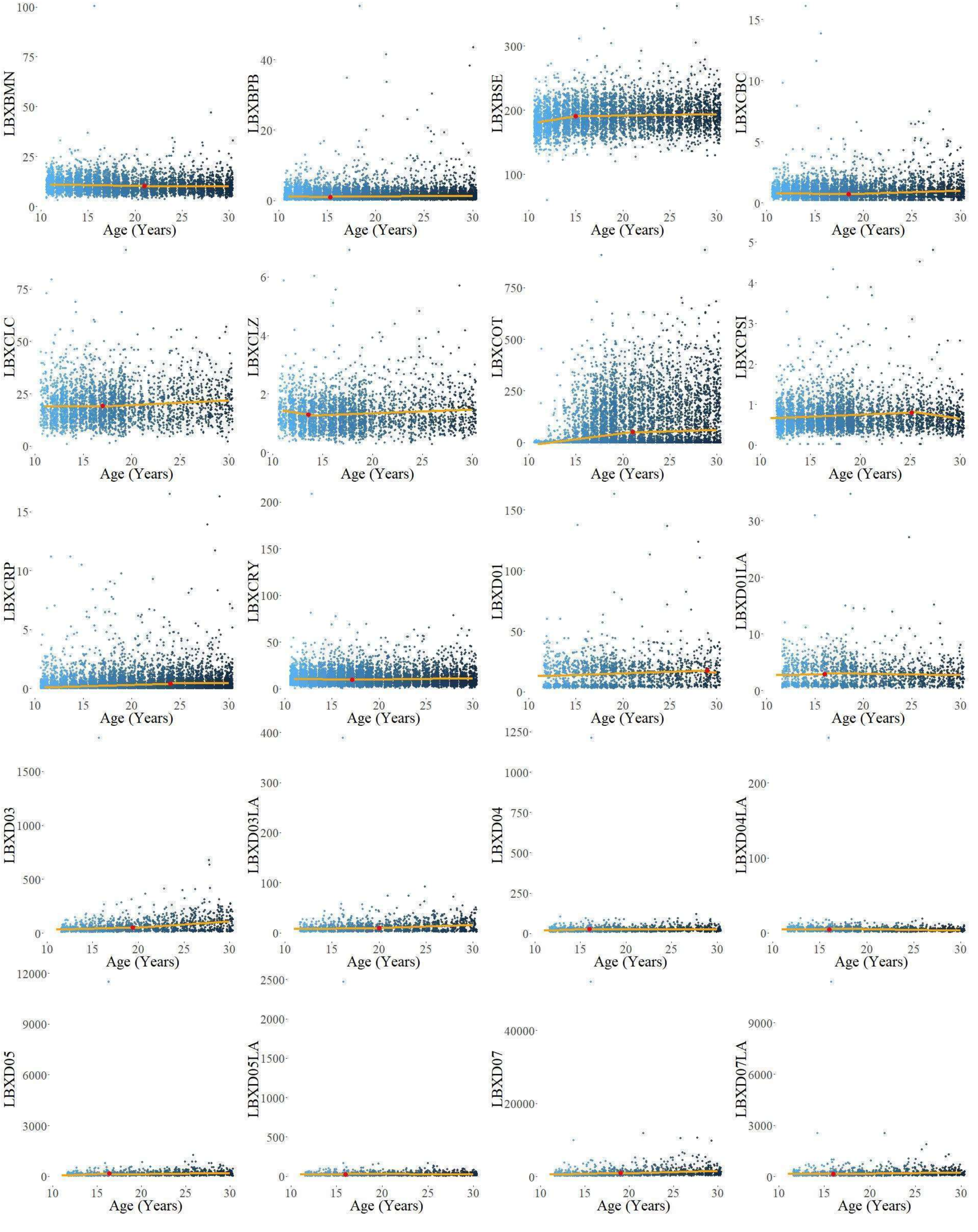
LBXBMN
Age (Years)
LBXBPB
Age (Years)
LBXBSE
Age (Years)
LBXCBC
Age (Years)
LBXCLC
Age (Years)
LBXCLZ
Age (Years)
LBXCOT
Age (Years)
LBXCPSI
Age (Years)
LBXCRP
Age (Years)
LBXCRY
Age (Years)
LBXD01
Age (Years)
LBXD01LA
Age (Years)
LBXD03
Age (Years)
LBXD03LA
Age (Years)
LBXD04
Age (Years)
LBXD04LA
Age (Years)
LBXD05
Age (Years)
LBXD05LA
Age (Years)
LBXD07
Age (Years)
LBXD07LA
Age (Years)

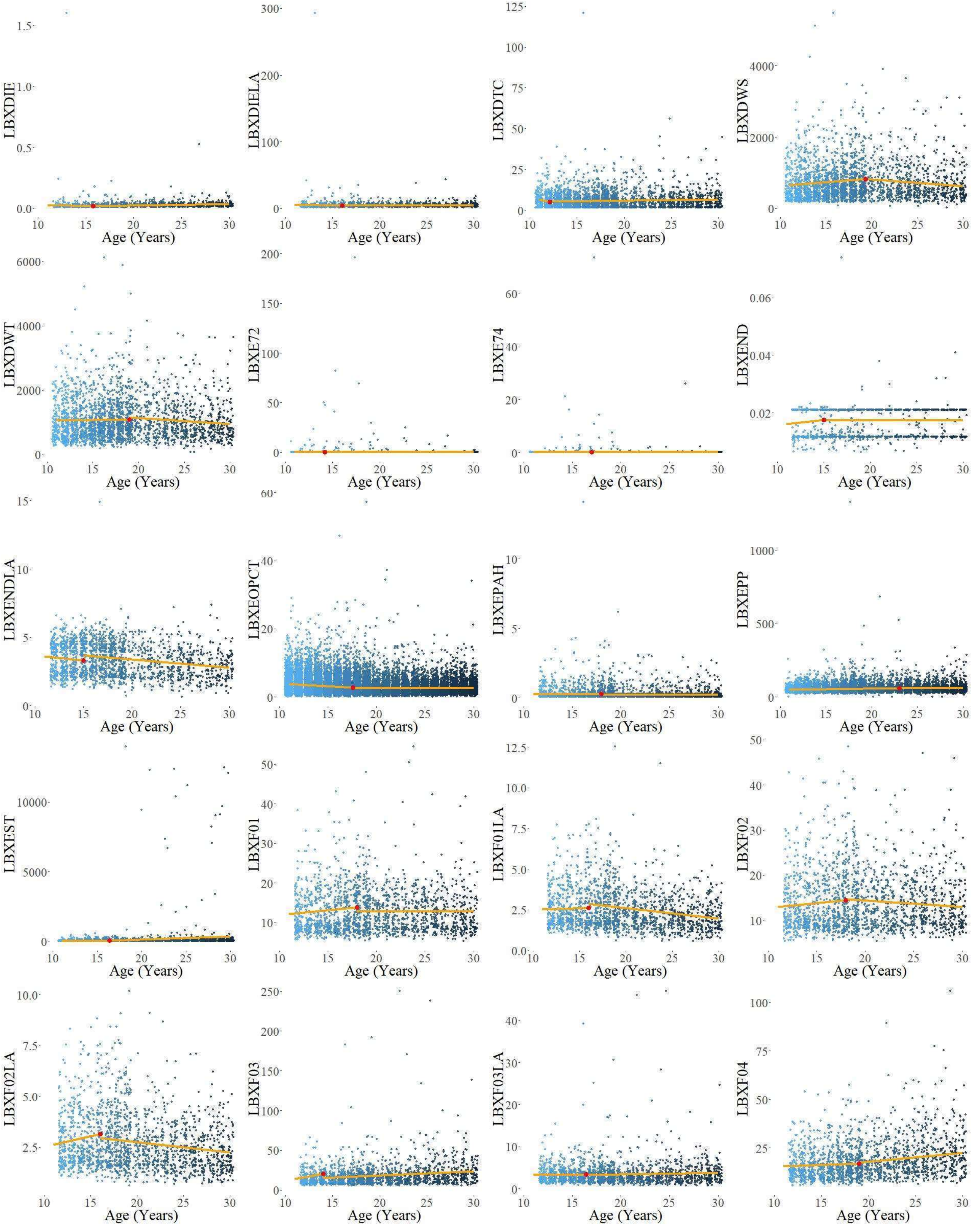

LBXDIE
LBXDIELA
LBXDTC
LBXDWS
LBXDWT
LBXE72
LBXE74
LBXEND
LBXENDLA
LBXEOPCT
LBXEPAH
LBXEPP
LBXEST
LBXF01
LBXF01LA
LBXF02
LBXF02LA
LBXF03
LBXF03LA
LBXF04
Age (Years)

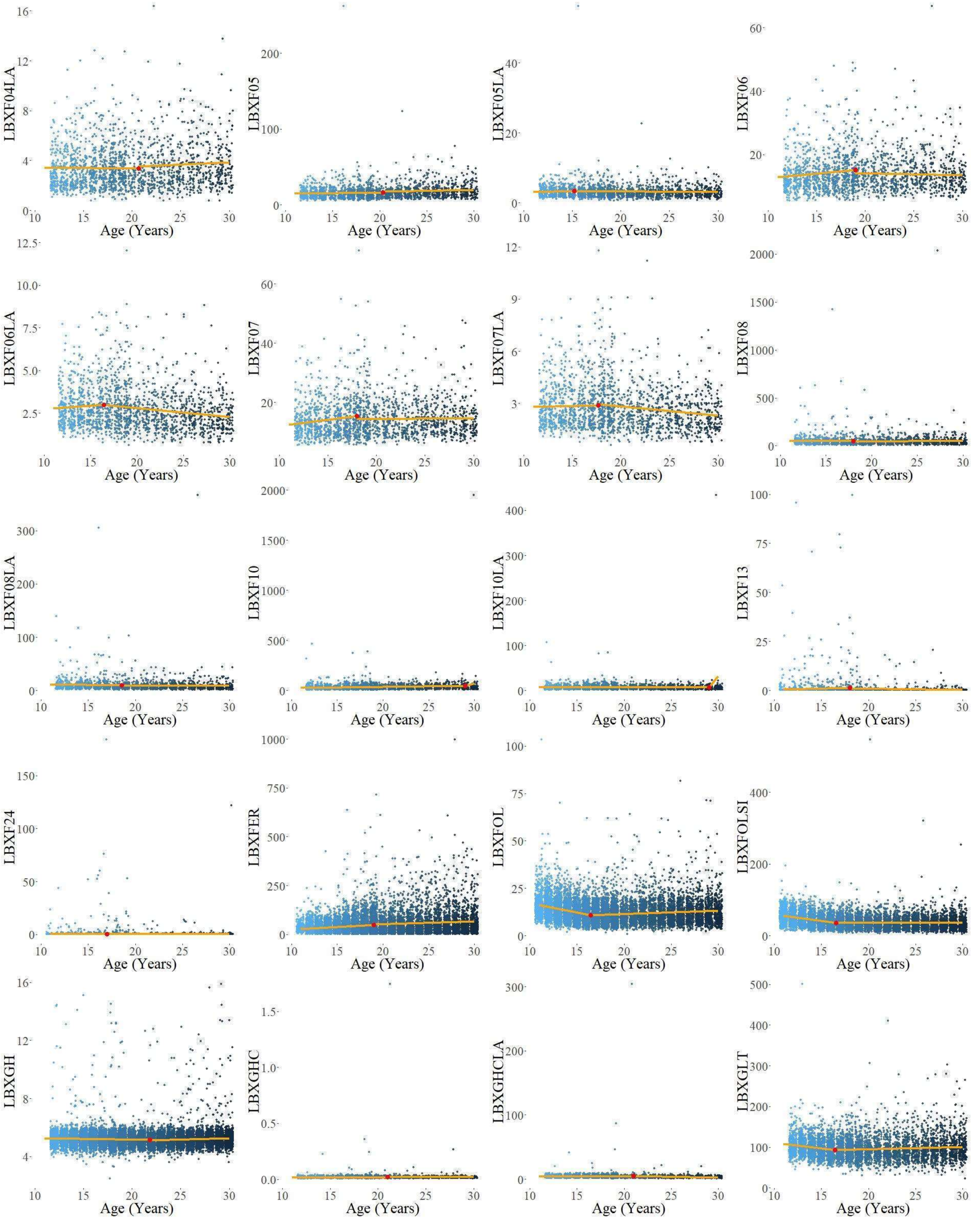

LBXF04LA
Age (Years)
LBXF05
Age (Years)
LBXF05LA
Age (Years)
LBXF06
Age (Years)
LBXF06LA
Age (Years)
LBXF07
Age (Years)
LBXF07LA
Age (Years)
LBXF08
Age (Years)
LBXF08LA
Age (Years)
LBXF10
Age (Years)
LBXF10LA
Age (Years)
LBXF13
Age (Years)
LBXF24
Age (Years)
LBXFER
Age (Years)
LBXFOL
Age (Years)
LBXFOLSI
Age (Years)
LBXGH
Age (Years)
LBXGHC
Age (Years)
LBXGHCLA
Age (Years)
LBXGLT
Age (Years)

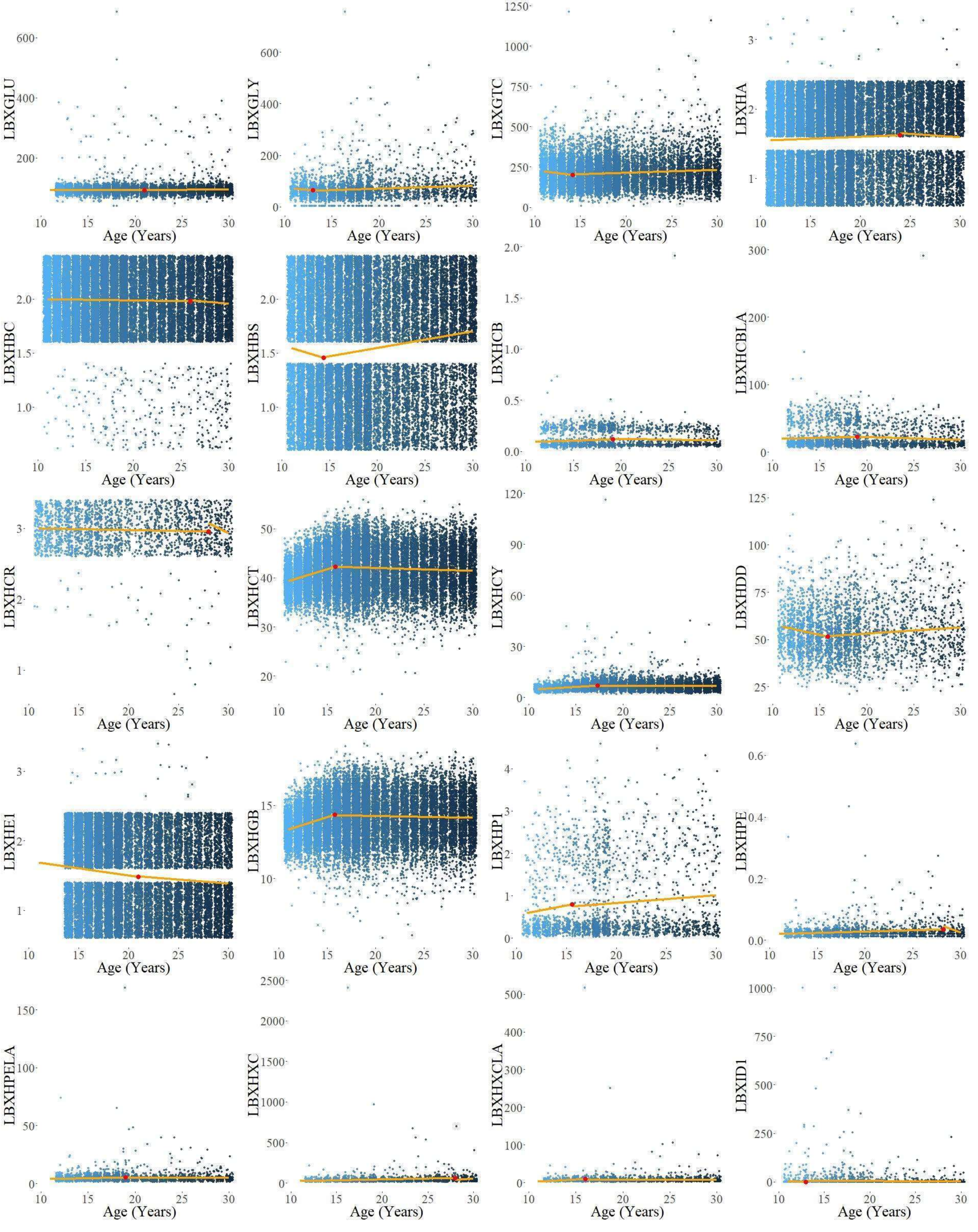

LBXGLU
LBXGLY
LBXGTC
LBXHA
LBXHBC
LBXHBS
LBXHCB
LBXHCBLA
LBXHCR
LBXHCT
LBXHCY
LBXHDD
LBXHE1
LBXHGB
LBXHP1
LBXHPE
LBXHPELA
LBXHXC
LBXHXCLA
LBXID1
Age (Years)

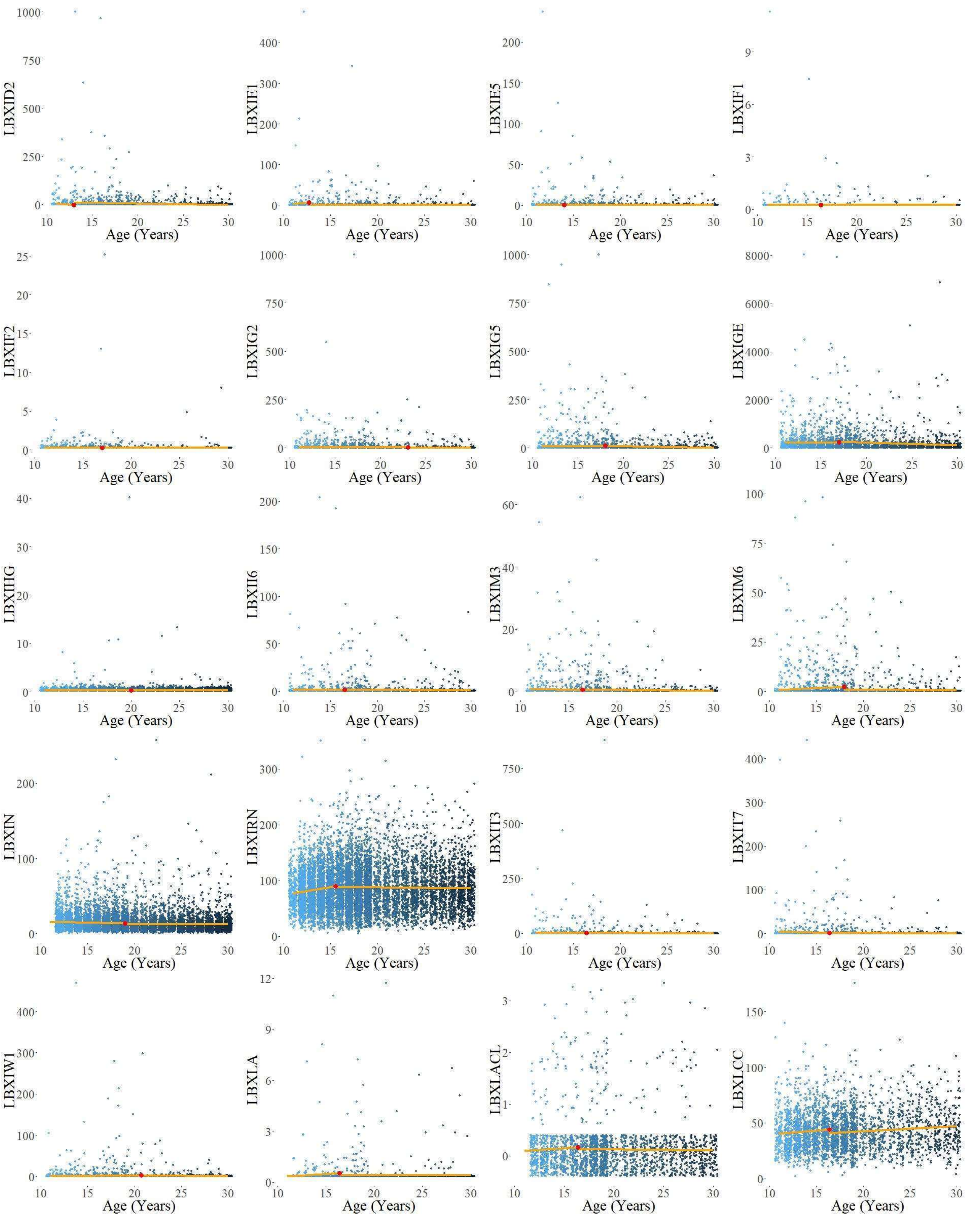

LBXID2
LBXIE1
LBXIE5
LBXIF1
LBXIF2
LBXIG2
LBXIG5
LBXIGE
LBXIHG
LBXII6
LBXIM3
LBXIM6
LBXIN
LBXIRN
LBXIT3
LBXIT7
LBXIW1
LBXLA
LBXLACL
LBXLCC
Age (Years)

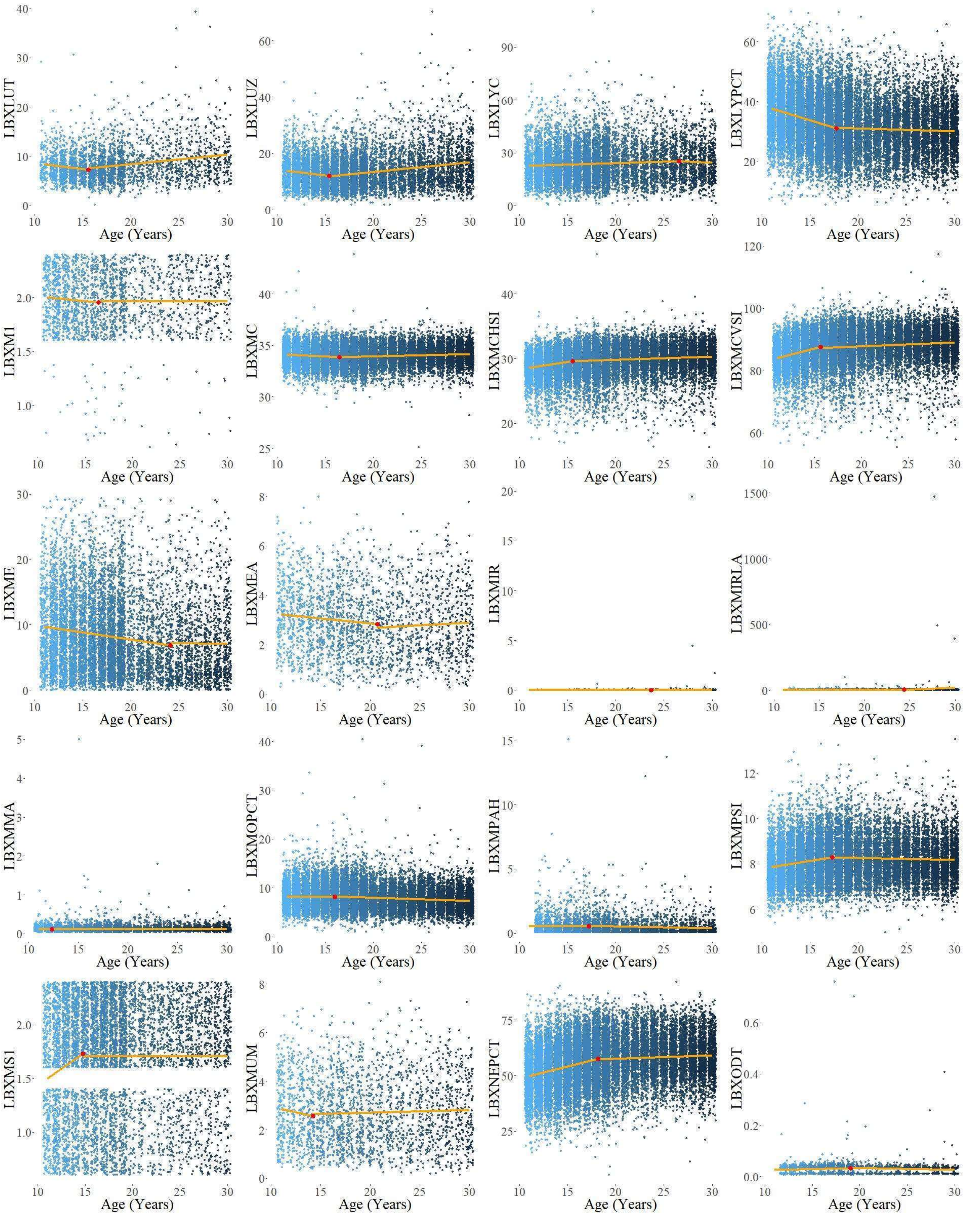

LBXLUT
Age (Years)
LBXLUZ
Age (Years)
LBXLYC
Age (Years)
LBXLYPCT
Age (Years)
LBXM1
Age (Years)
LBXMC
Age (Years)
LBXMCHSI
Age (Years)
LBXMCVSI
Age (Years)
LBXME
Age (Years)
LBXMEA
Age (Years)
LBXMIR
Age (Years)
LBXMIRLA
Age (Years)
LBXMMA
Age (Years)
LBXMOPCT
Age (Years)
LBXMPAH
Age (Years)
LBXMPSI
Age (Years)
LBXMS1
Age (Years)
LBXMUM
Age (Years)
LBXNEPCT
Age (Years)
LBXODT
Age (Years)

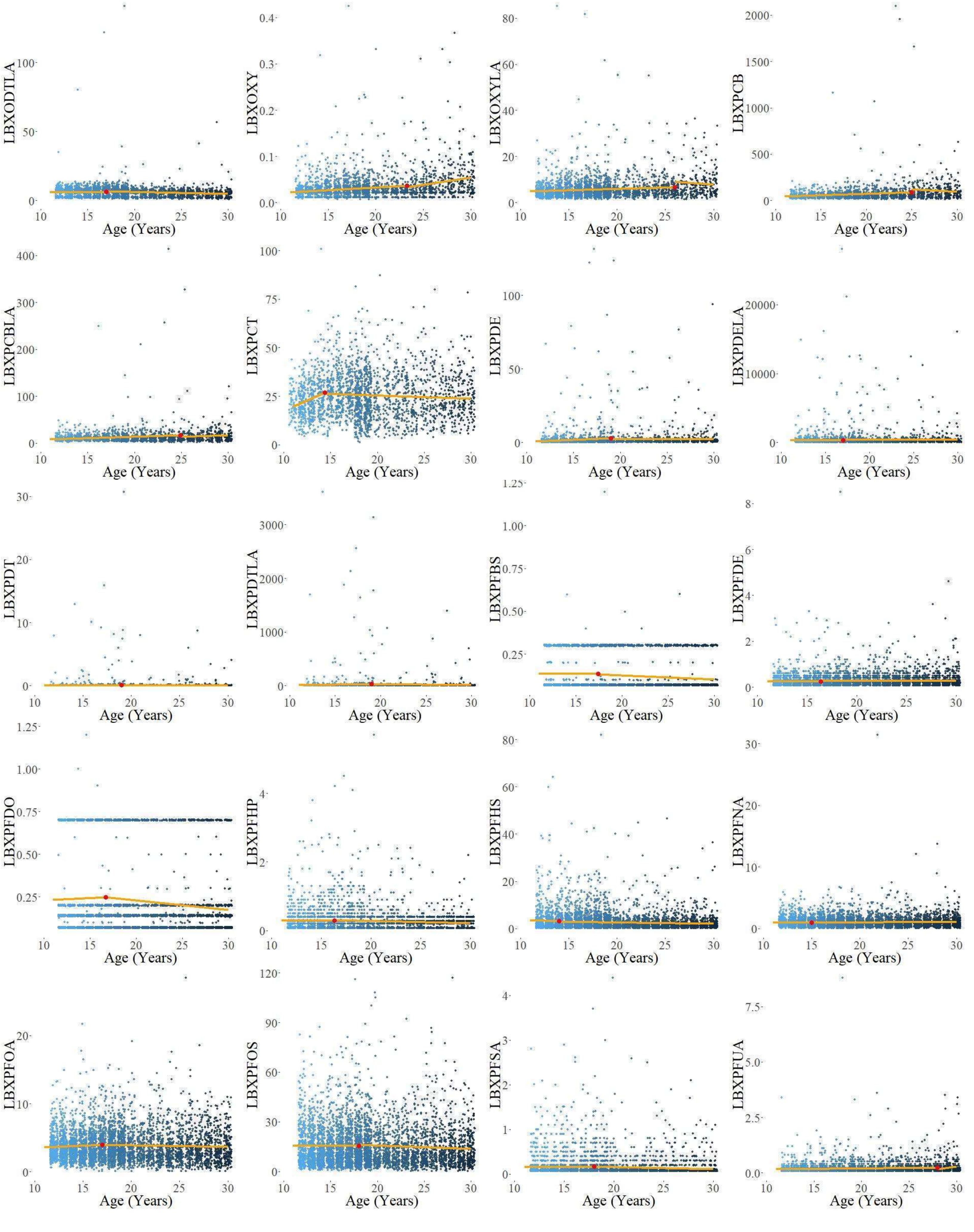
LBXODTLA
LBXOXY
LBXOXYLA
LBXPCB
LBXPCBLA
LBXPCT
LBXPDE
LBXPDELA
LBXPDT
LBXPDTLA
LBXPFBS
LBXPFDE
LBXPFDO
LBXPFHP
LBXPFHS
LBXPFNA
LBXPFOA
LBXPFOS
LBXPFSA
LBXPFUA
Age (Years)

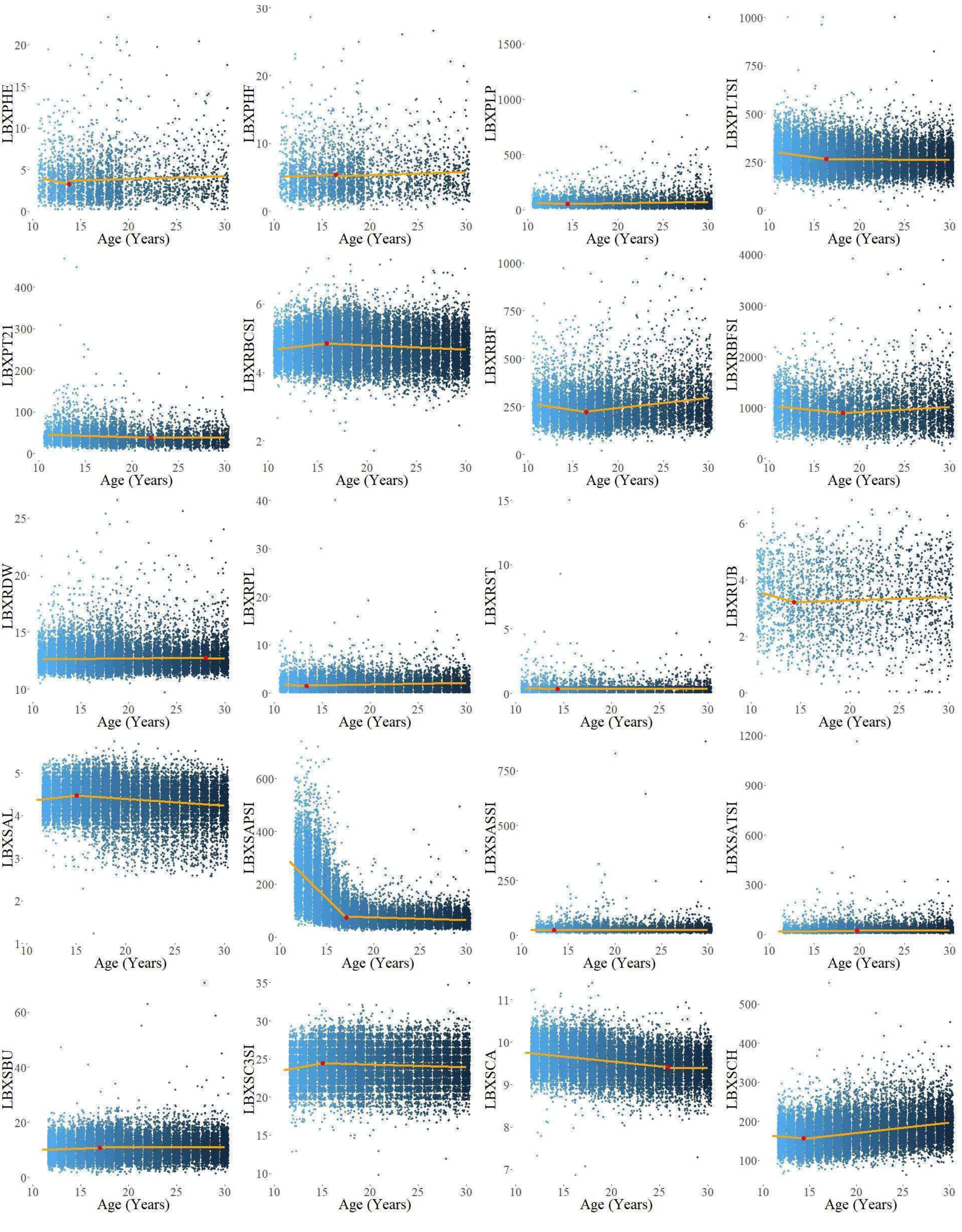

LBXPHE
LBXPHF
LBXPLP
LBXPLTSI
LBXPT21
LBXRBCSI
LBXRBF
LBXRBFSI
LBXRDW
LBXRPL
LBXRST
LBXRUB
LBXSAL
LBXSAPSI
LBXSASSI
LBXSATSI
LBXSBU
LBXSC3SI
LBXSCA
LBXSCH
Age (Years)

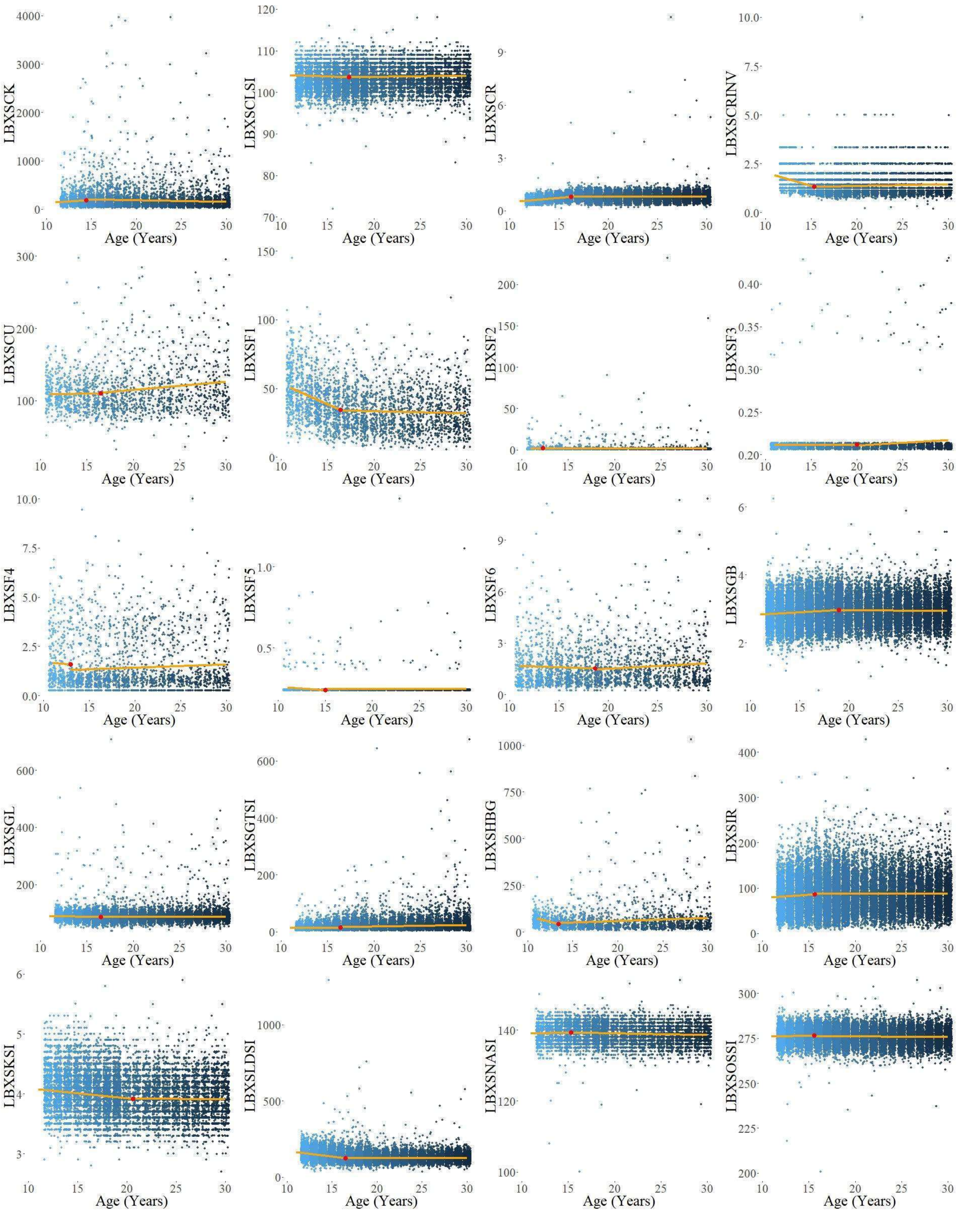

LBXSCK
LBXSCLSI
LBXSCR
LBXSCRINV
LBXSCU
LBXSF1
LBXSF2
LBXSF3
LBXSF4
LBXSF5
LBXSF6
LBXSGB
LBXSGL
LBXSGTSI
LBXSHBG
LBXSIR
LBXSKSI
LBXSLDSI
LBXSNASI
LBXSOSSI
Age (Years)

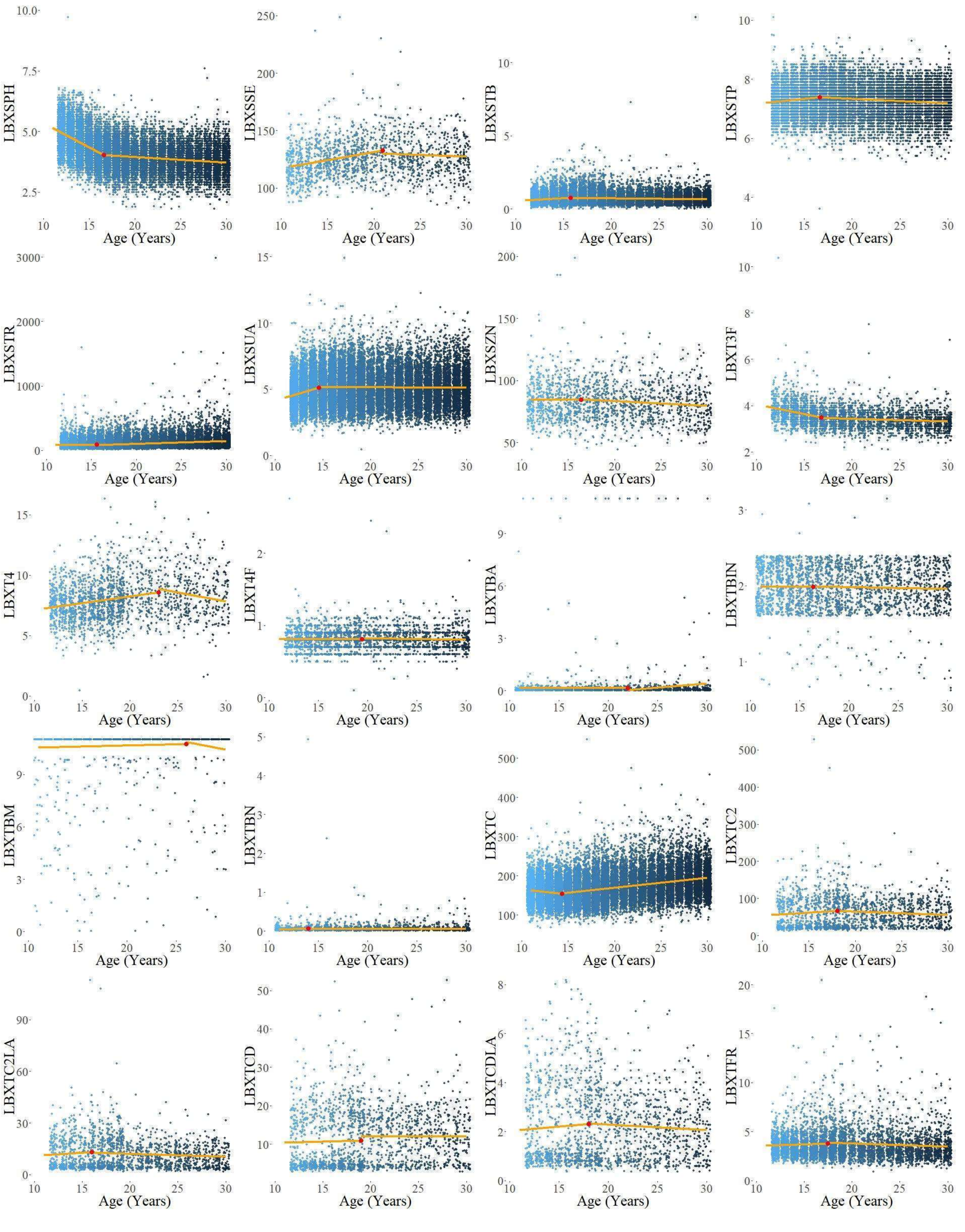

LBXSPH
Age (Years)
LBXSSE
Age (Years)
LBXSTB
Age (Years)
LBXSTP
Age (Years)
LBXSTR
Age (Years)
LBXSUA
Age (Years)
LBXSZN
Age (Years)
LBXT3F
Age (Years)
LBXT4
Age (Years)
LBXT4F
Age (Years)
LBXTBA
Age (Years)
LBXTBIN
Age (Years)
LBXTBM
Age (Years)
LBXTBN
Age (Years)
LBXTC
Age (Years)
LBXTC2
Age (Years)
LBXTC2LA
Age (Years)
LBXTCD
Age (Years)
LBXTCDLA
Age (Years)
LBXTFR
Age (Years)

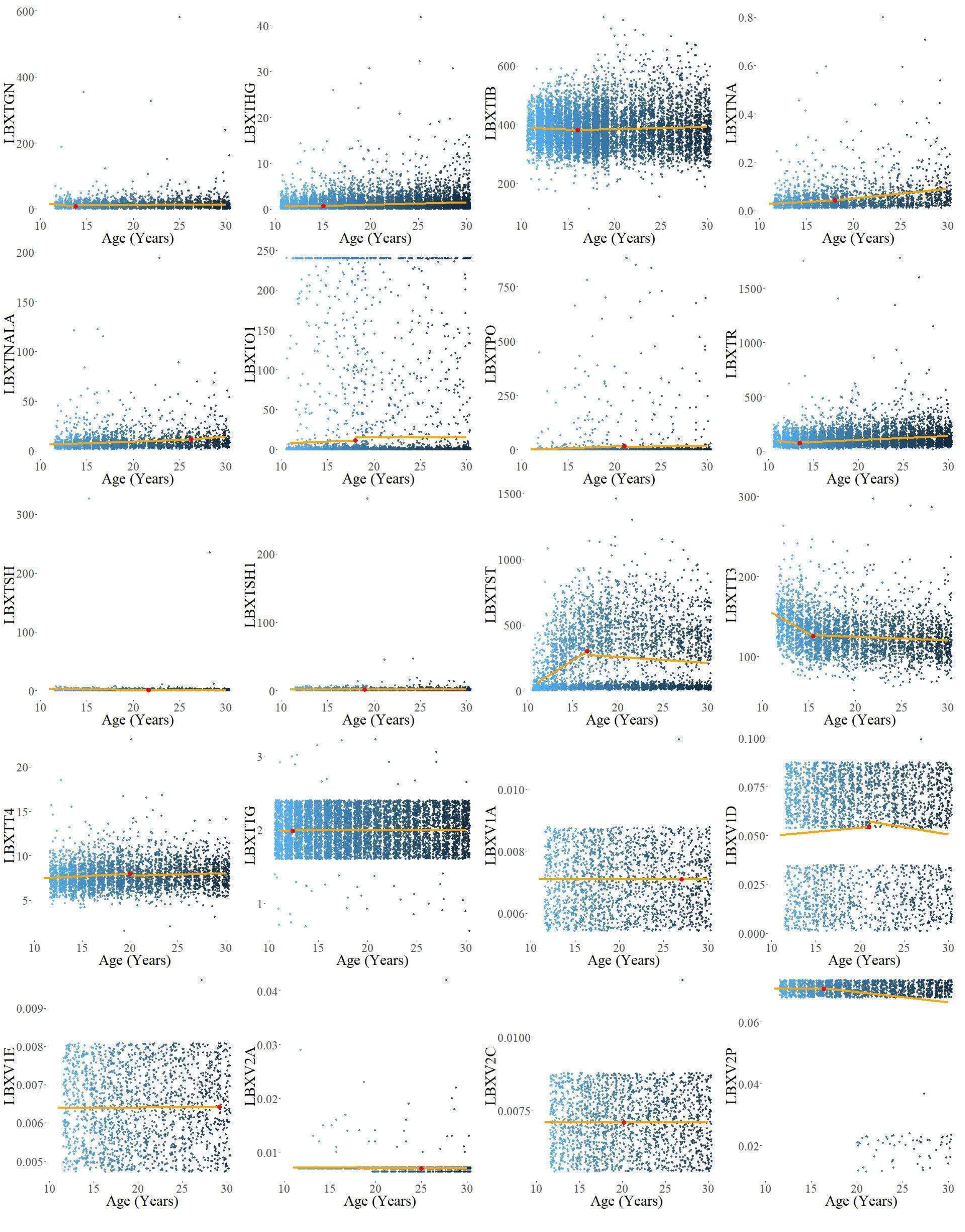
LBXTGN
Age (Years)
LBXTHG
Age (Years)
LBXTIB
Age (Years)
LBXTNA
Age (Years)
LBXTNALA
Age (Years)
LBXTO1
Age (Years)
LBXTPO
Age (Years)
LBXTR
Age (Years)
LBXTSH
Age (Years)
LBXTSH1
Age (Years)
LBXTST
Age (Years)
LBXTT3
Age (Years)
LBXTT4
Age (Years)
LBXTTG
Age (Years)
LBXV1A
Age (Years)
LBXV1D
Age (Years)
LBXV1E
Age (Years)
LBXV2A
Age (Years)
LBXV2C
Age (Years)
LBXV2P
Age (Years)

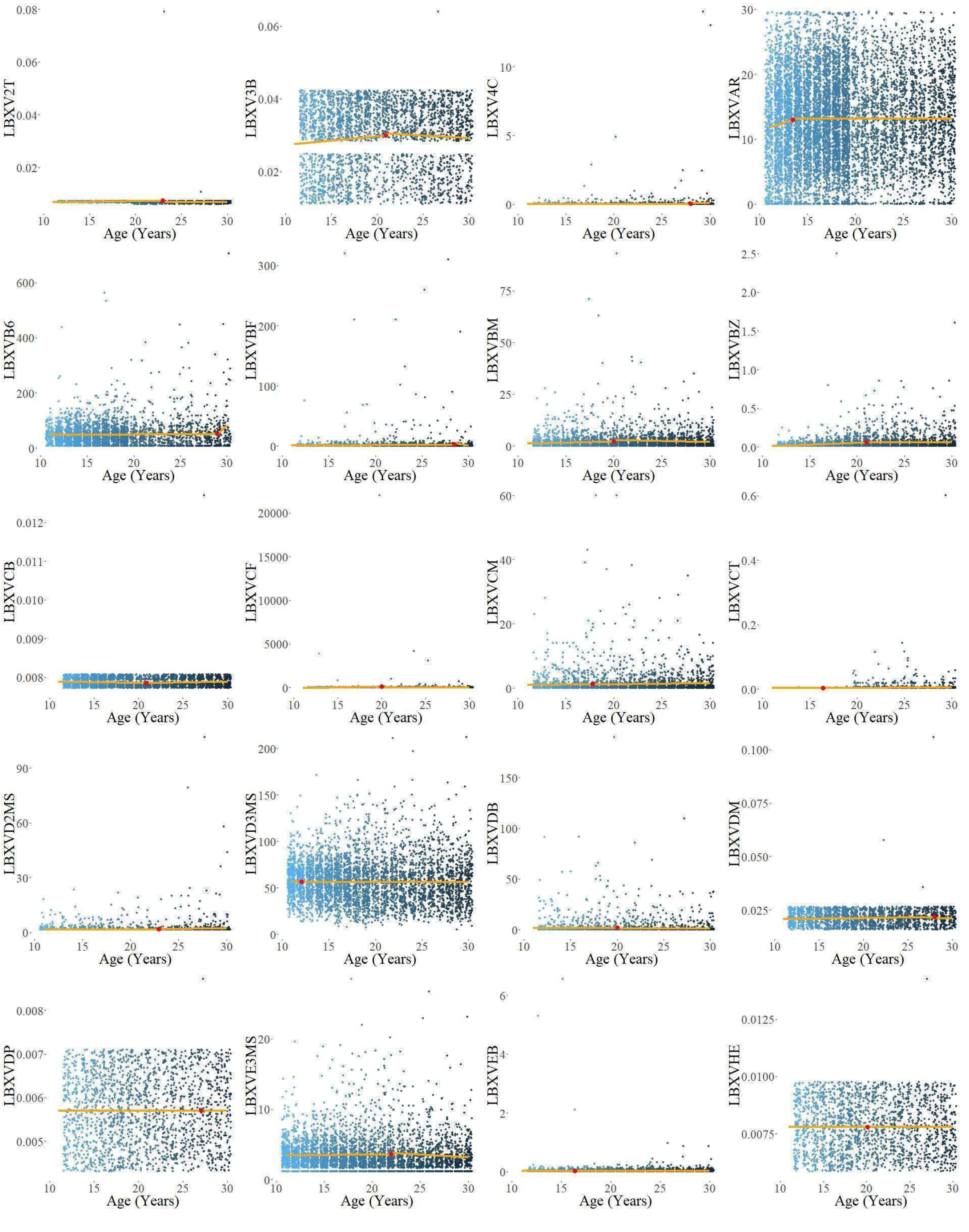
LBXV2T
Age (Years)
LBXV3B
Age (Years)
LBXV4C
Age (Years)
LBXVAR
Age (Years)
LBXVB6
Age (Years)
LBXVBF
Age (Years)
LBXVBM
Age (Years)
LBXVBZ
Age (Years)
LBXVCB
Age (Years)
LBXVCF
Age (Years)
LBXVCM
Age (Years)
LBXVCT
Age (Years)
LBXVD2MS
Age (Years)
LBXVD3MS
Age (Years)
LBXVDB
Age (Years)
LBXVDM
Age (Years)
LBXVDP
Age (Years)
LBXVE3MS
Age (Years)
LBXVEB
Age (Years)
LBXVHE
Age (Years)

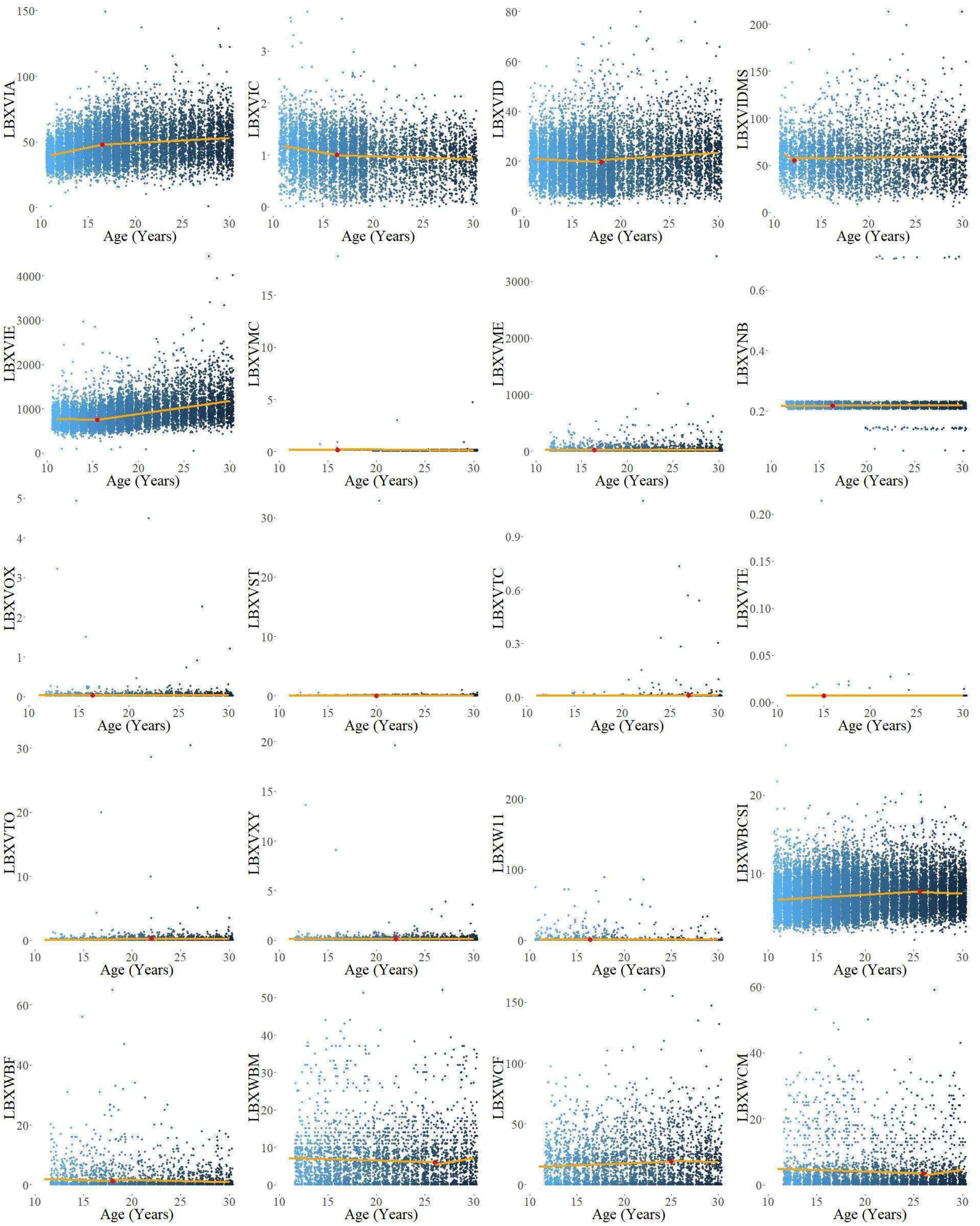

LBXVIA
0
50
100
150
LBXVIC
0
1
2
3
LBXVID
0
20
40
60
80
LBXVIDMS
0
50
100
150
200
LBXVIE
0
1000
2000
3000
4000
LBXVMC
0
5
10
15
LBXVME
0
1000
2000
3000
LBXVNB
0.2
0.4
0.6
LBXVOX
0
1
2
3
4
5
LBXVST
0
10
20
30
LBXVTC
0.0
0.3
0.6
0.9
LBXVTE
0.00
0.05
0.10
0.15
0.20
LBXVTO
0
10
20
30
LBXVXY
0
5
10
15
20
LBXW11
0
100
200
LBXWBCSI
10
20
LBXWBF
0
20
40
60
LBXWBM
0
10
20
30
40
50
LBXWCF
0
50
100
150
LBXWCM
0
20
40
60
10
15
20
25
30
Age (Years)

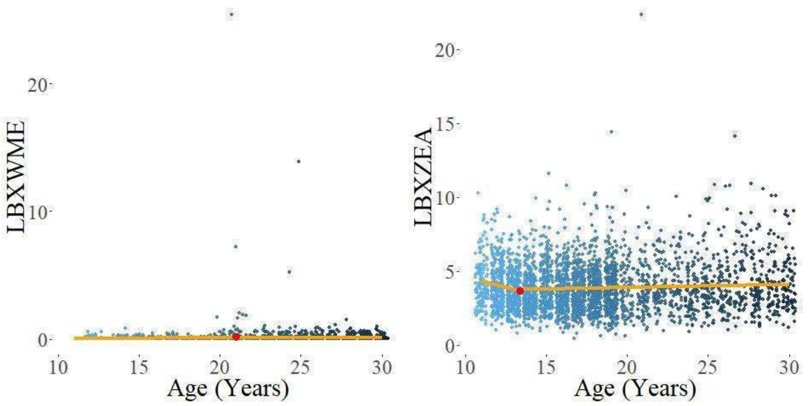

LBXWME
20
10
0
10
15
20
25
30
Age (Years)
LBXZEA
20
15
10
5
0
10
15
20
25
30
Age (Years)

Supplementary Figure 2. Analyte levels by age, colored by gender for 301 analytes.

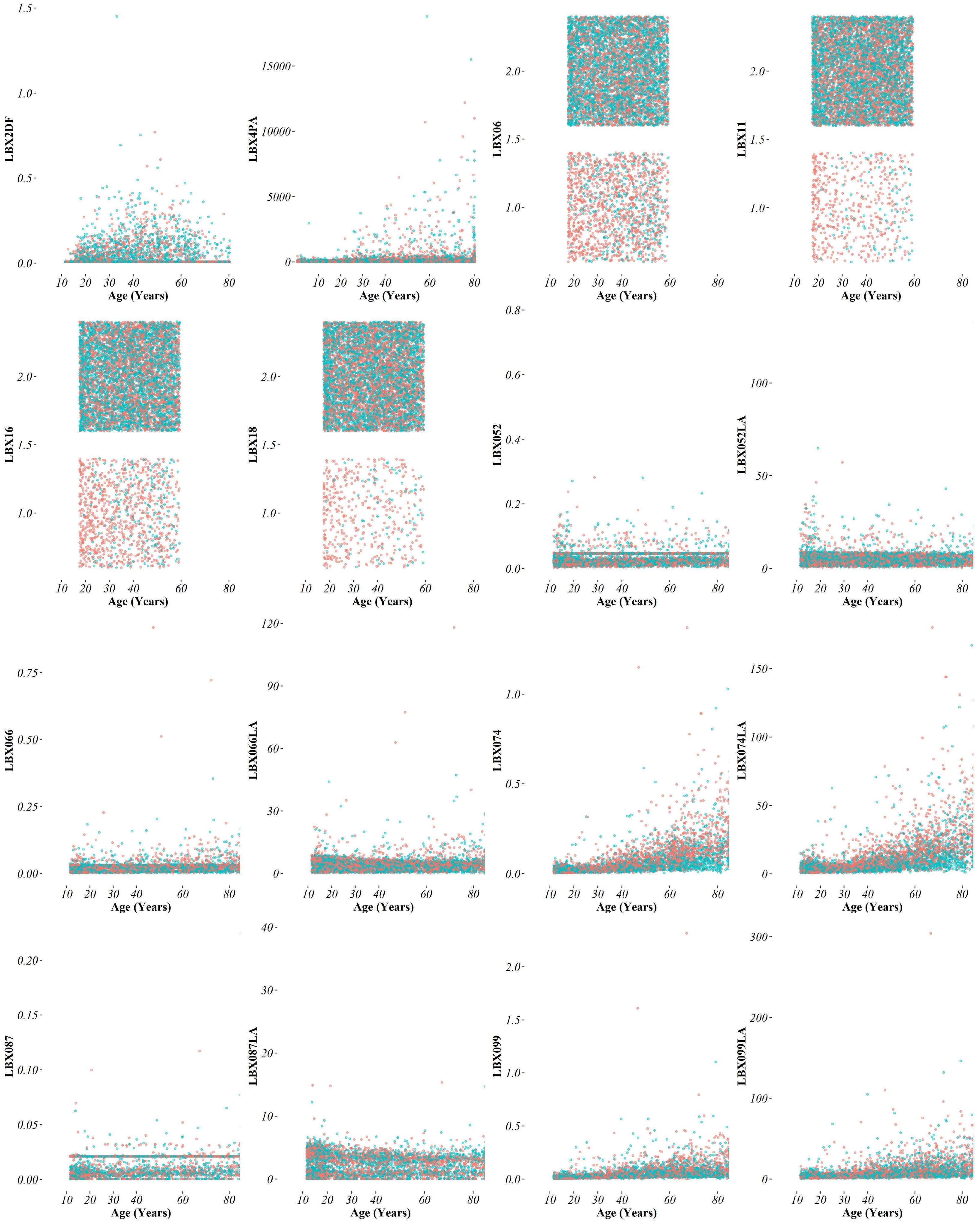
LBX2DF
LBX4PA
LBX06
LBX11
LBX16
LBX18
LBX052
LBX052LA
LBX066
LBX066LA
LBX074
LBX074LA
LBX087
LBX087LA
LBX099
LBX099LA
Age (Years)

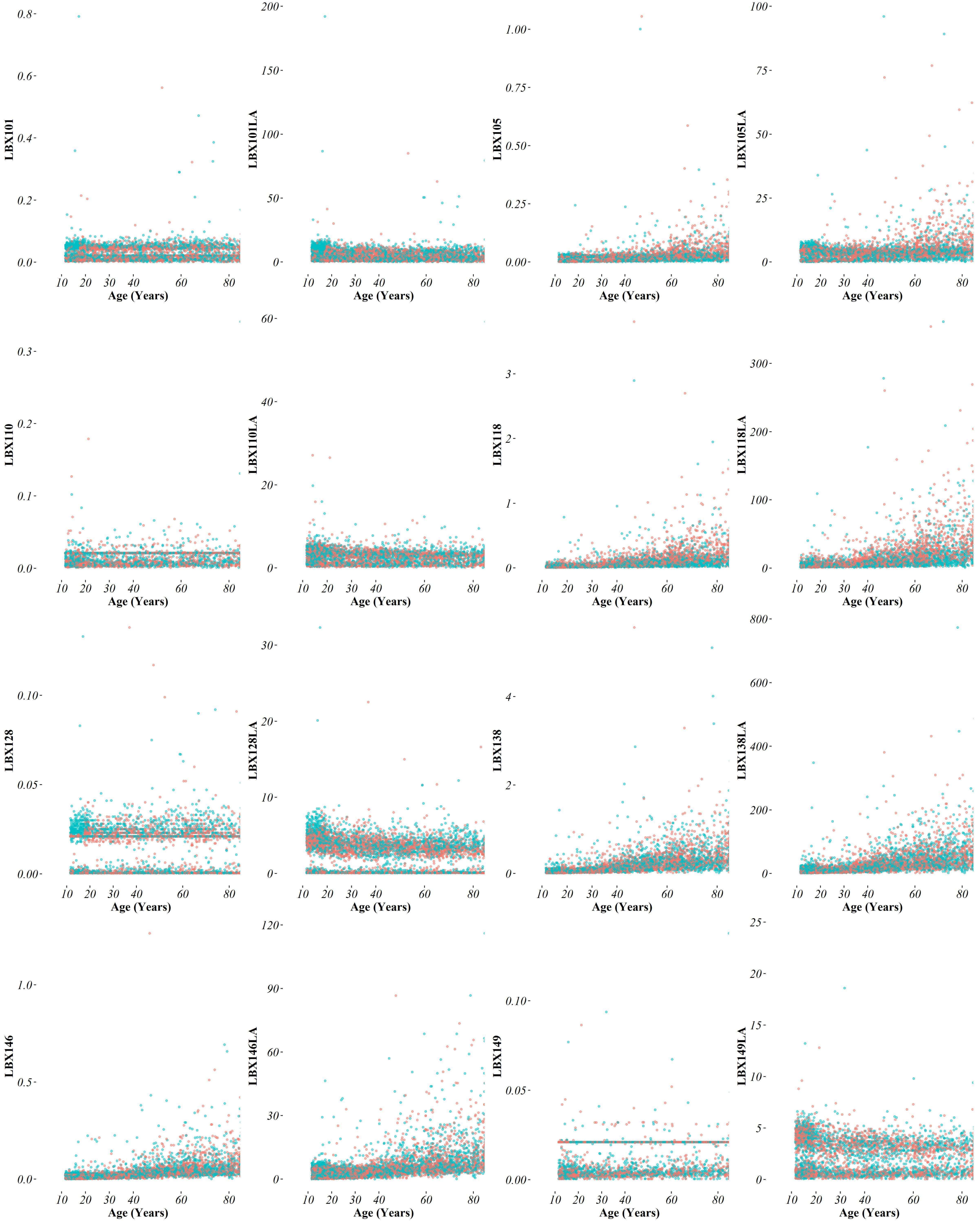
LBX101
Age (Years)
LBX101LA
Age (Years)
LBX105
Age (Years)
LBX105LA
Age (Years)
LBX110
Age (Years)
LBX110LA
Age (Years)
LBX118
Age (Years)
LBX118LA
Age (Years)
LBX128
Age (Years)
LBX128LA
Age (Years)
LBX138
Age (Years)
LBX138LA
Age (Years)
LBX146
Age (Years)
LBX146LA
Age (Years)
LBX149
Age (Years)
LBX149LA
Age (Years)

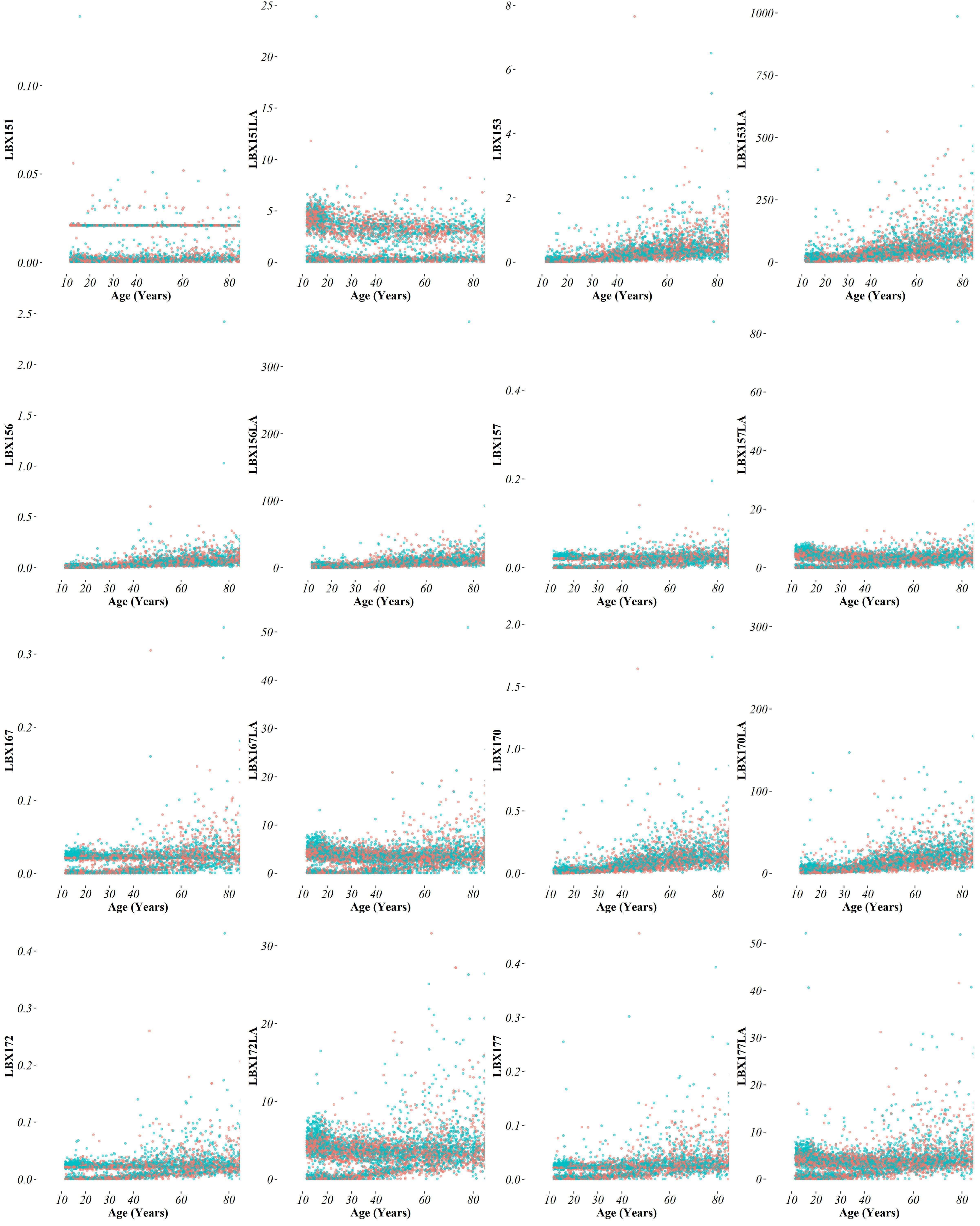
LBX151
LBX151LA
LBX153
LBX153LA
LBX156
LBX156LA
LBX157
LBX157LA
LBX167
LBX167LA
LBX170
LBX170LA
LBX172
LBX172LA
LBX177
LBX177LA
Age (Years)

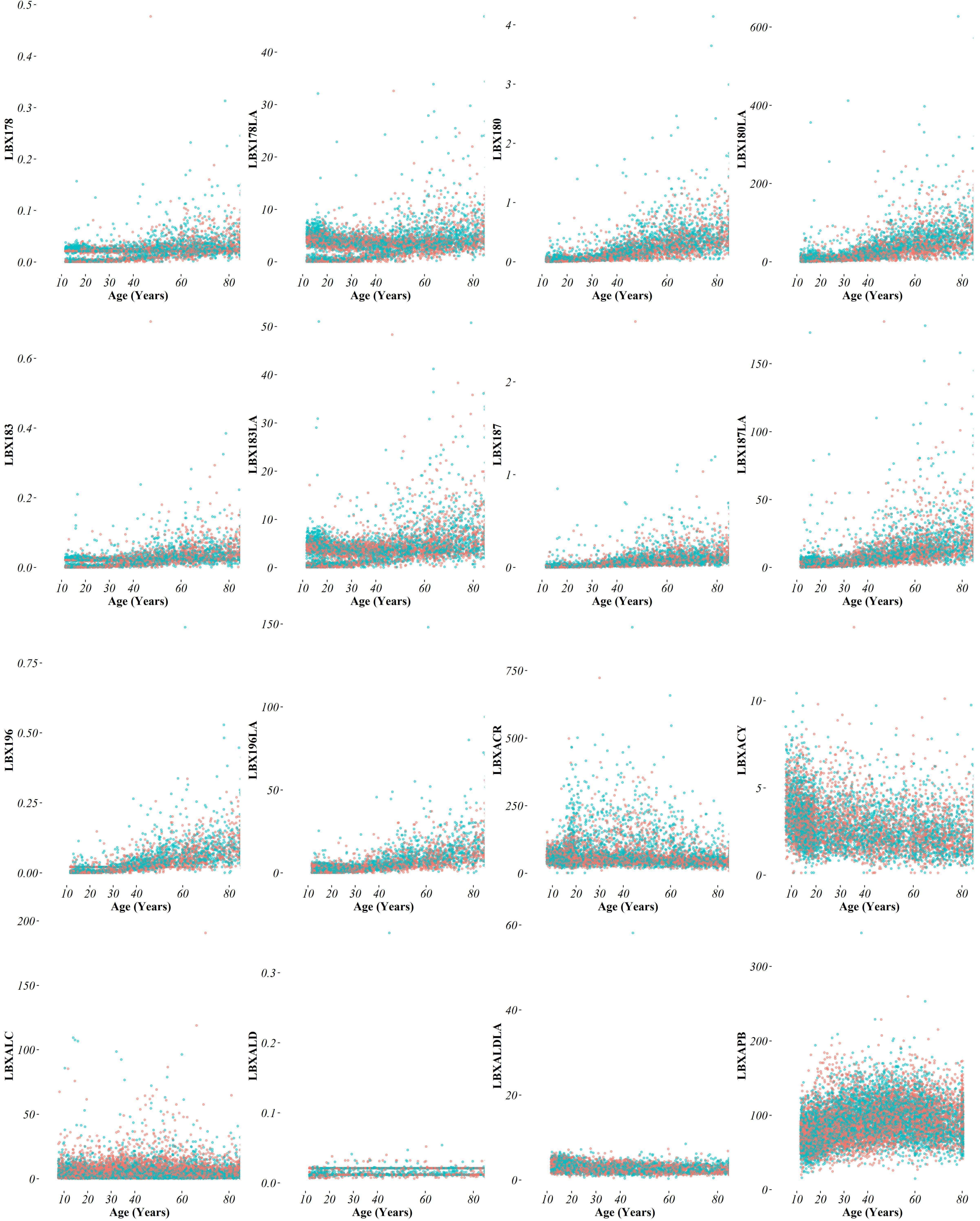
LBX178
LBX178LA
LBX180
LBX180LA
LBX183
LBX183LA
LBX187
LBX187LA
LBX196
LBX196LA
LBXACR
LBXACY
LBXALC
LBXALD
LBXALDLA
LBXAPB
Age (Years)

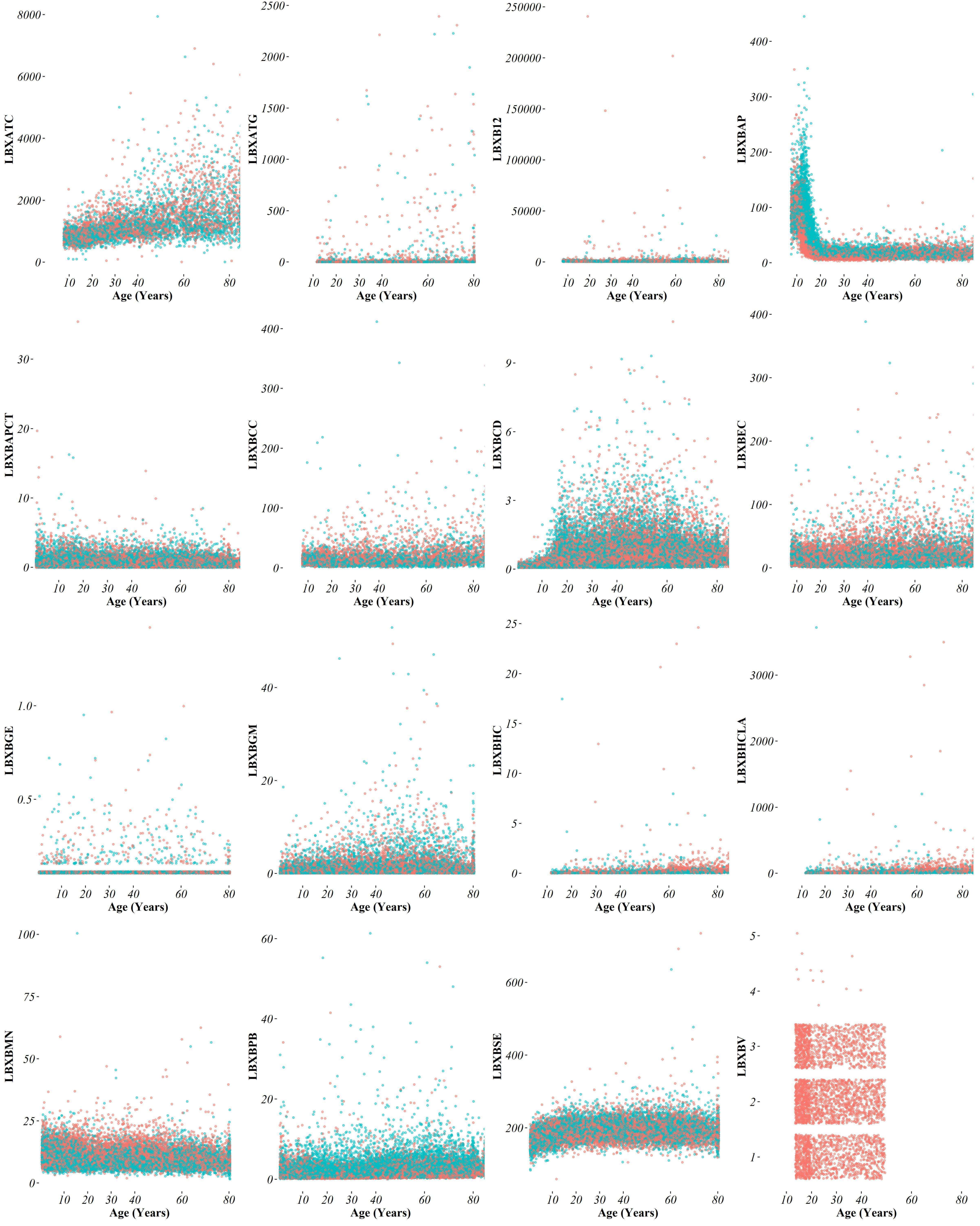

LBXATC
LBXATG
LBXB12
LBXBAP
LBXBAPCT
LBXBCC
LBXBCD
LBXBEC
LBXBGE
LBXBGM
LBXBHC
LBXBHCLA
LBXBMN
LBXBPB
LBXBSE
LBXBV
Age (Years)

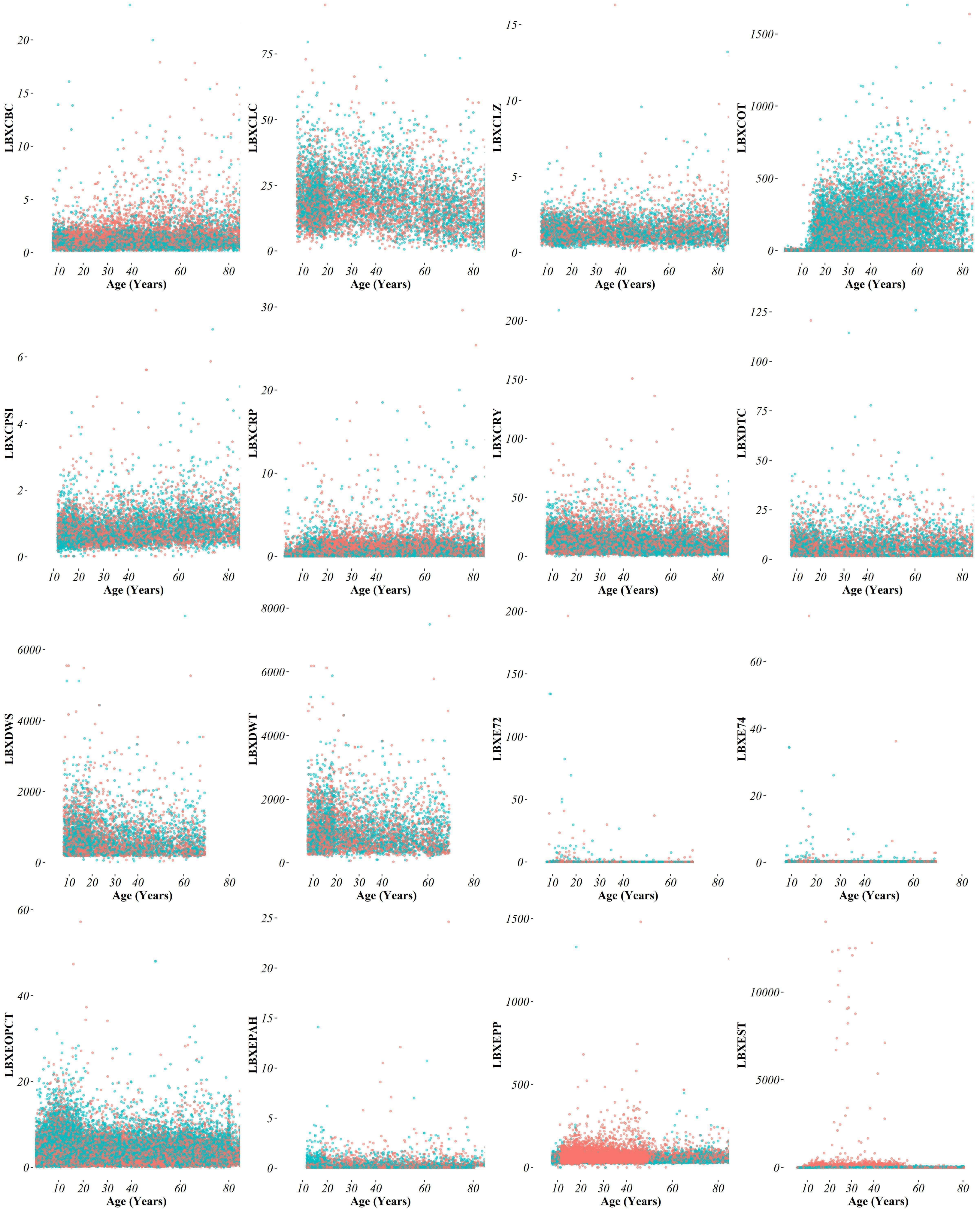

LBXCBC
LBXCLC
LBXCLZ
LBXCOT
LBXCPSI
LBXCRP
LBXCRY
LBXDTC
LBXDWS
LBXDWT
LBXE72
LBXE74
LBXEOPCT
LBXEPAH
LBXEPP
LBXEST
Age (Years)

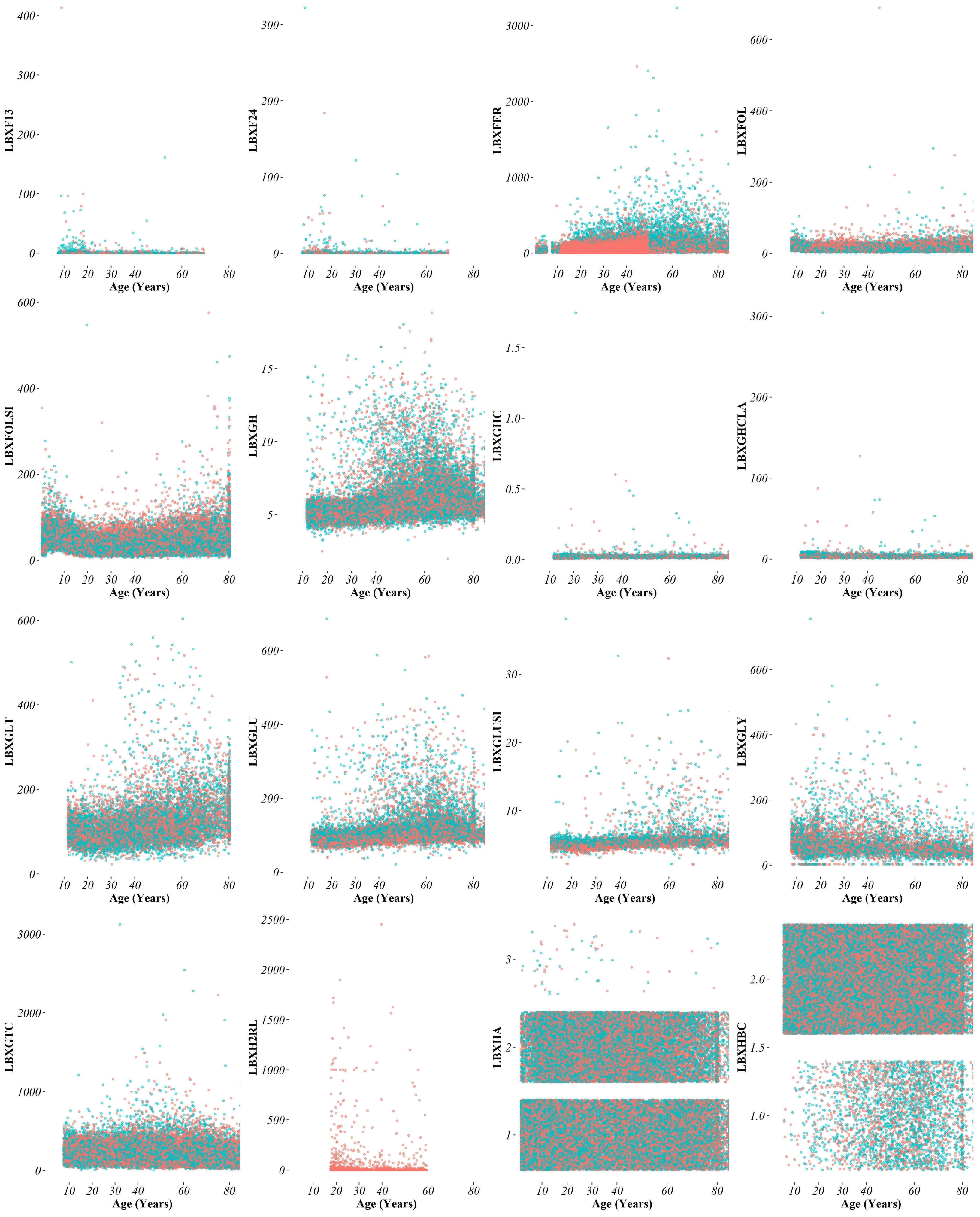
LBXF13
LBXF24
LBXFER
LBXFOL
LBXFOLSI
LBXGH
LBXGHC
LBXGHCLA
LBXGLT
LBXGLU
LBXGLUSI
LBXGLY
LBXGTC
LBXH2RL
LBXHA
LBXHBC
Age (Years)

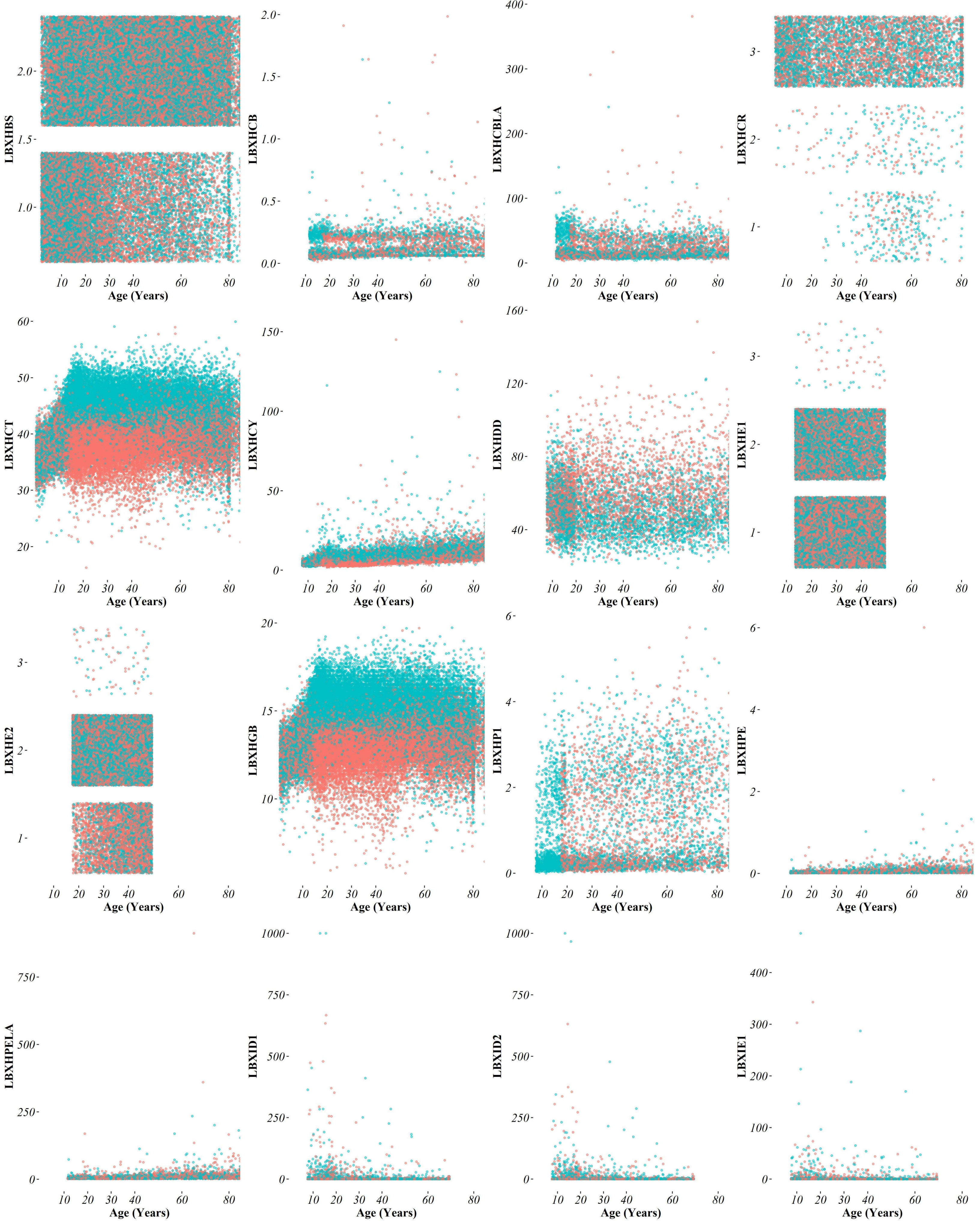
LBXHBS
Age (Years)
LBXHCB
Age (Years)
LBXHCBLA
Age (Years)
LBXHCR
Age (Years)
LBXHCT
Age (Years)
LBXHCY
Age (Years)
LBXHDD
Age (Years)
LBXHE1
Age (Years)
LBXHE2
Age (Years)
LBXHGB
Age (Years)
LBXHP1
Age (Years)
LBXHPE
Age (Years)
LBXHPELA
Age (Years)
LBXID1
Age (Years)
LBXID2
Age (Years)
LBXIE1
Age (Years)

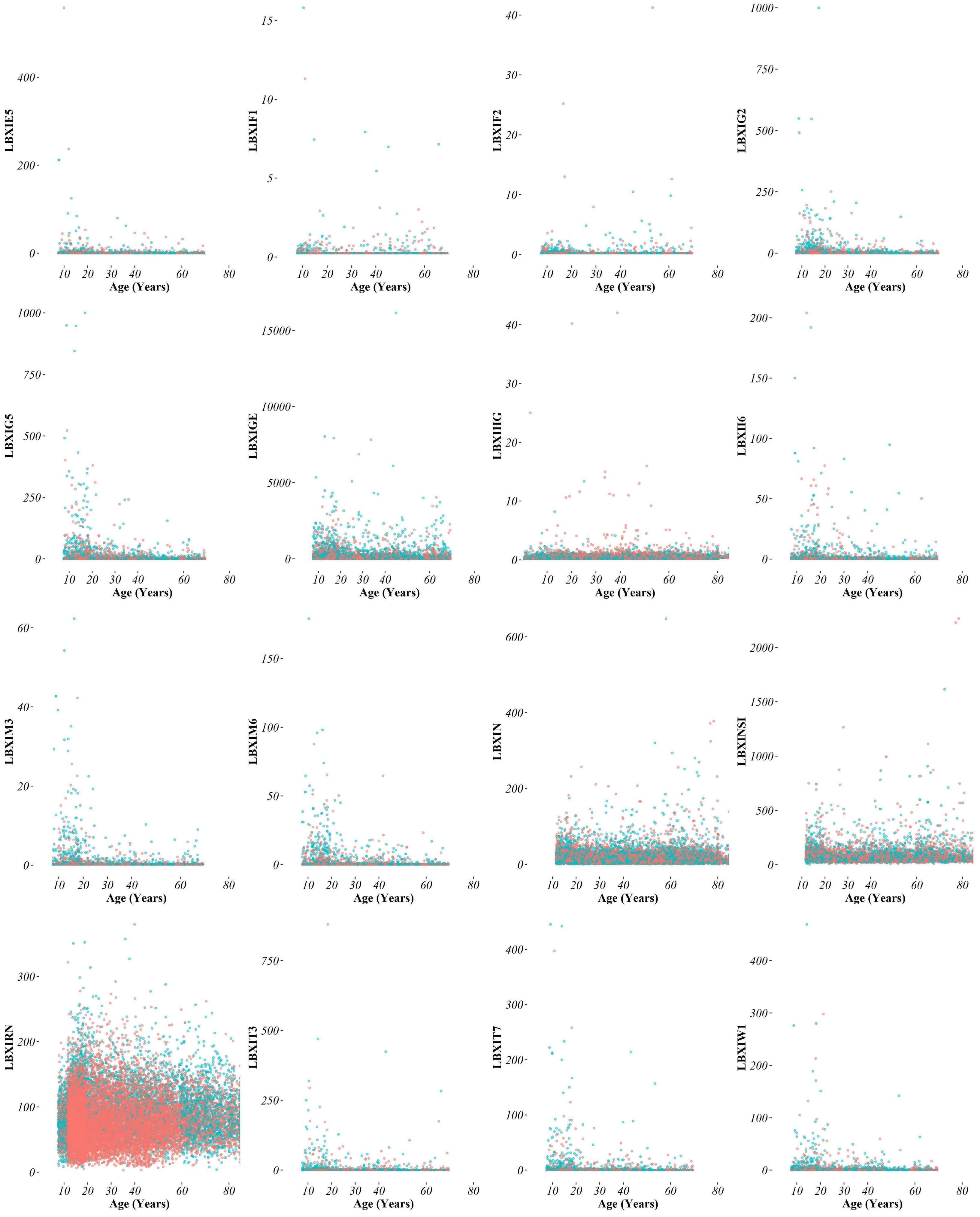

LBXIE5
0
200
400
LBXIF1
0
5
10
15
LBXIF2
0
10
20
30
40
LBXIG2
0
250
500
750
1000
LBXIG5
0
250
500
750
1000
LBXIGE
0
5000
10000
15000
LBXIHG
0
10
20
30
40
LBXII6
0
50
100
150
200
LBXIM3
0
20
40
60
LBXIM6
0
50
100
150
LBXIN
0
200
400
600
LBXINSI
0
500
1000
1500
2000
LBXIRN
0
100
200
300
LBXIT3
0
250
500
750
LBXIT7
0
100
200
300
400
LBXIW1
0
100
200
300
400
10 20 30 40 60 80
Age (Years)

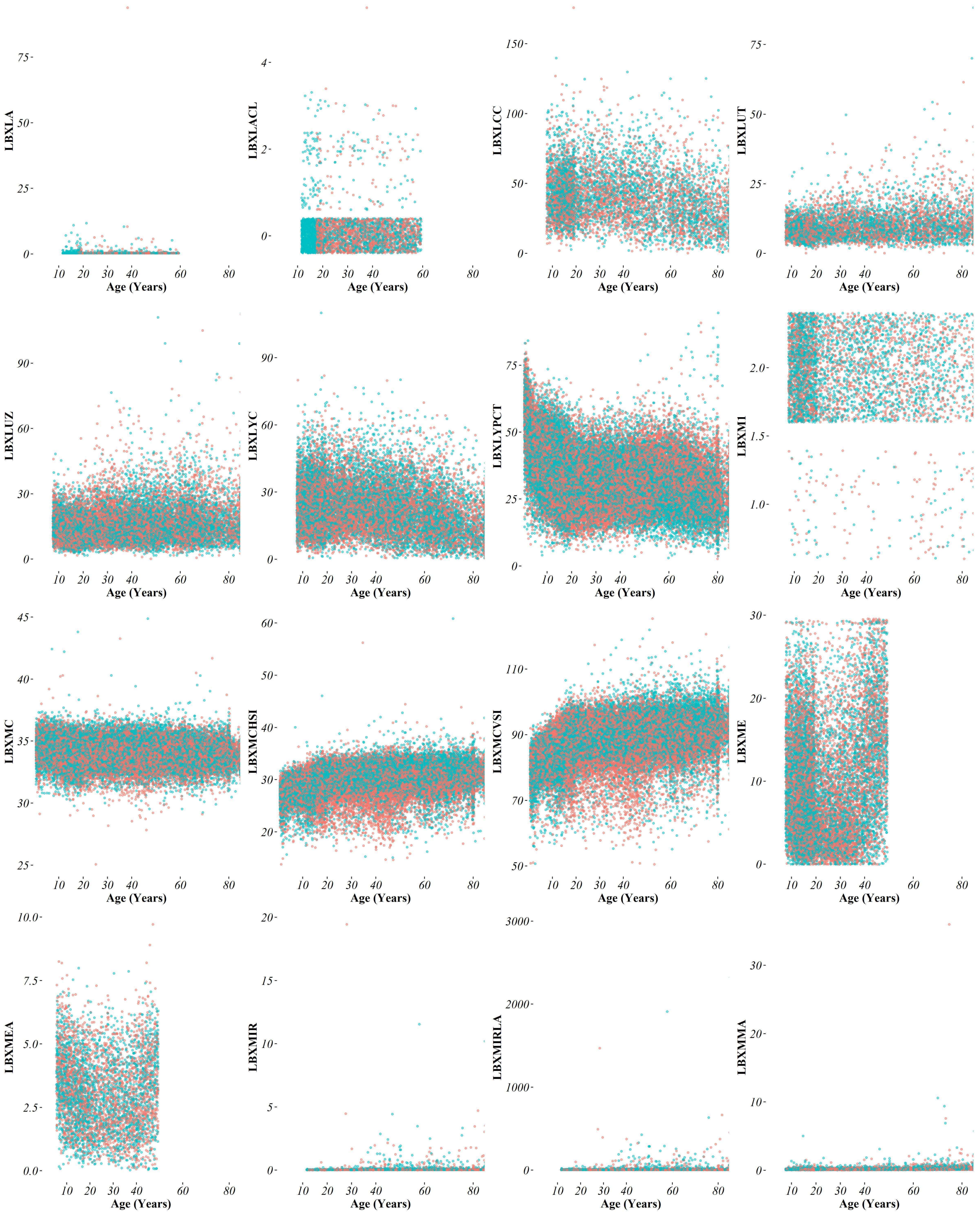
LBXLA
0
25
50
75
10 20 30 40 60 80
Age (Years)
LBXLACL
0
2
4
10 20 30 40 60 80
Age (Years)
LBXLCC
0
50
100
150
10 20 30 40 60 80
Age (Years)
LBXLUT
0
25
50
75
10 20 30 40 60 80
Age (Years)
LBXLUZ
0
30
60
90
10 20 30 40 60 80
Age (Years)
LBXLYC
0
30
60
90
10 20 30 40 60 80
Age (Years)
LBXLYPCT
0
25
50
75
10 20 30 40 60 80
Age (Years)
LBXM1
1.0
1.5
2.0
10 20 30 40 60 80
Age (Years)
LBXMC
25
30
35
40
45
10 20 30 40 60 80
Age (Years)
LBXMCHSI
20
30
40
50
60
10 20 30 40 60 80
Age (Years)
LBXMCVSI
50
70
90
110
10 20 30 40 60 80
Age (Years)
LBXME
0
10
20
30
10 20 30 40 60 80
Age (Years)
LBXMEA
0.0
2.5
5.0
7.5
10.0
10 20 30 40 60 80
Age (Years)
LBXMIR
0
5
10
15
20
10 20 30 40 60 80
Age (Years)
LBXMIRLA
0
1000
2000
3000
10 20 30 40 60 80
Age (Years)
LBXMMA
0
10
20
30
10 20 30 40 60 80
Age (Years)

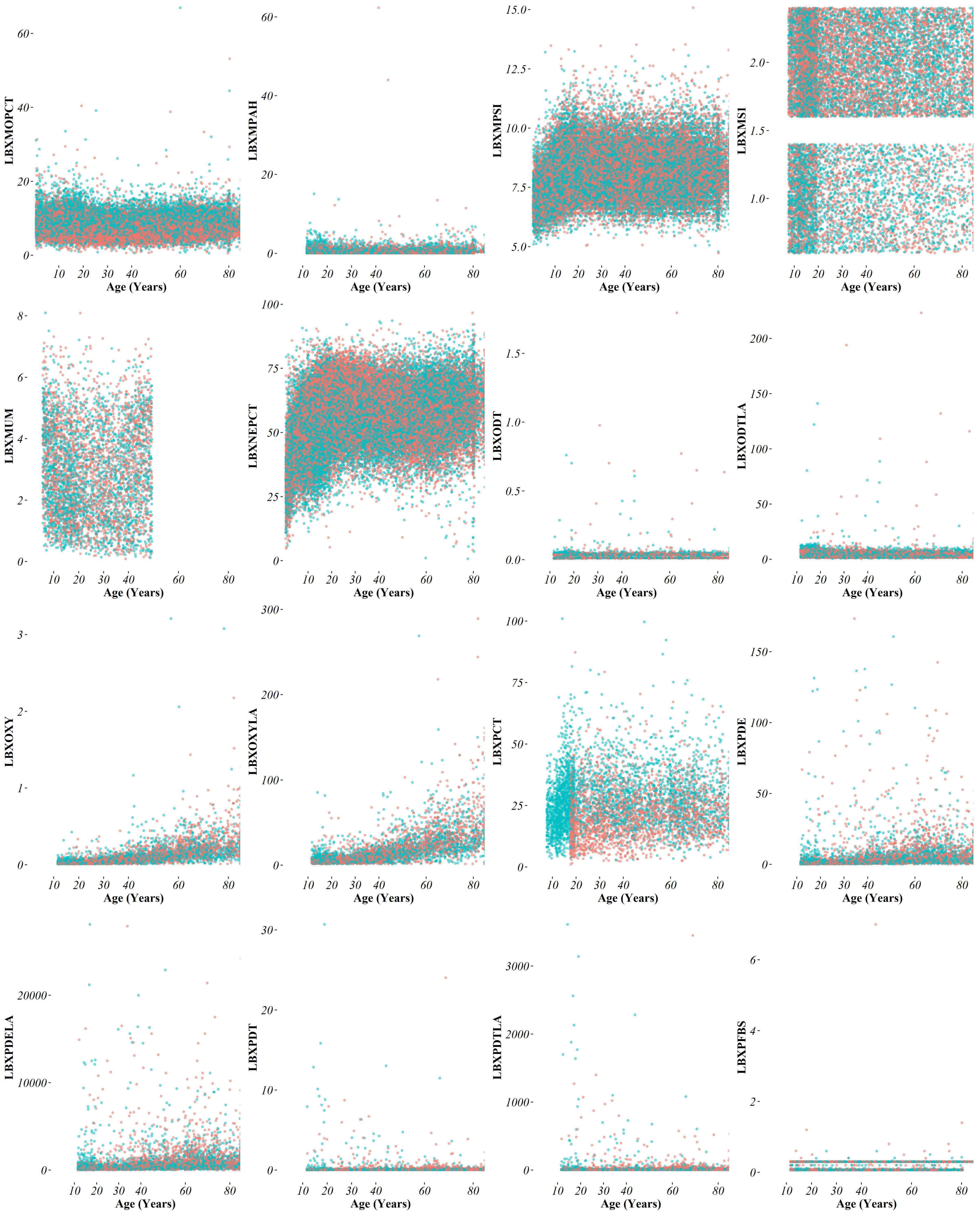
LBXMOPCT
LBXMPAH
LBXMPSI
LBXMS1
LBXMUM
LBXNEPCT
LBXODT
LBXODTLA
LBXOXY
LBXOXYLA
LBXPCT
LBXPDE
LBXPDELA
LBXPDT
LBXPDTLA
LBXPFBS
Age (Years)

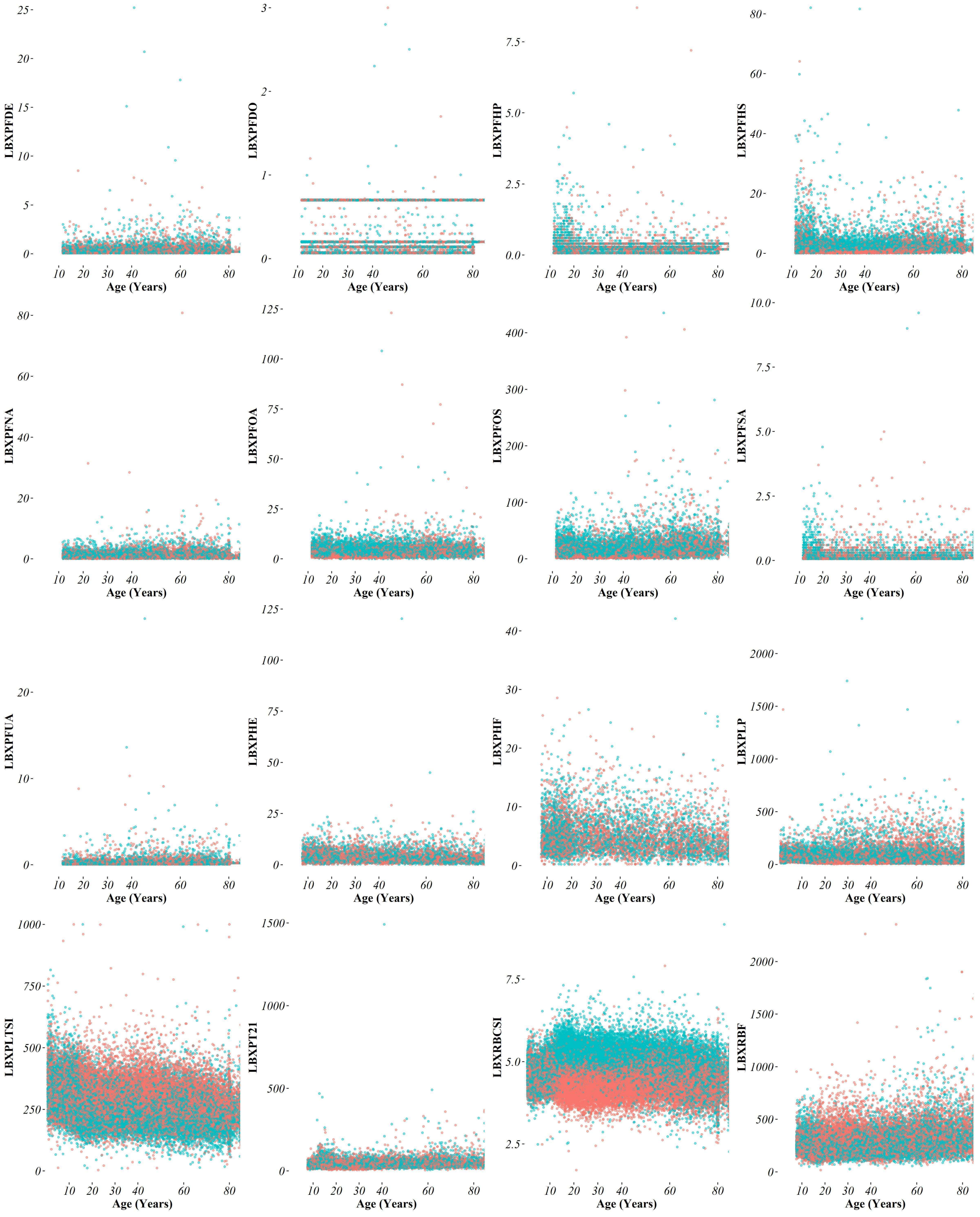
LBXPFDE
LBXPFDO
LBXPFHP
LBXPFHS
LBXPFNA
LBXPFOA
LBXPFOS
LBXPFSA
LBXPFUA
LBXPHE
LBXPHF
LBXPLP
LBXPLTSI
LBXPT21
LBXRBCSI
LBXRBF
Age (Years)

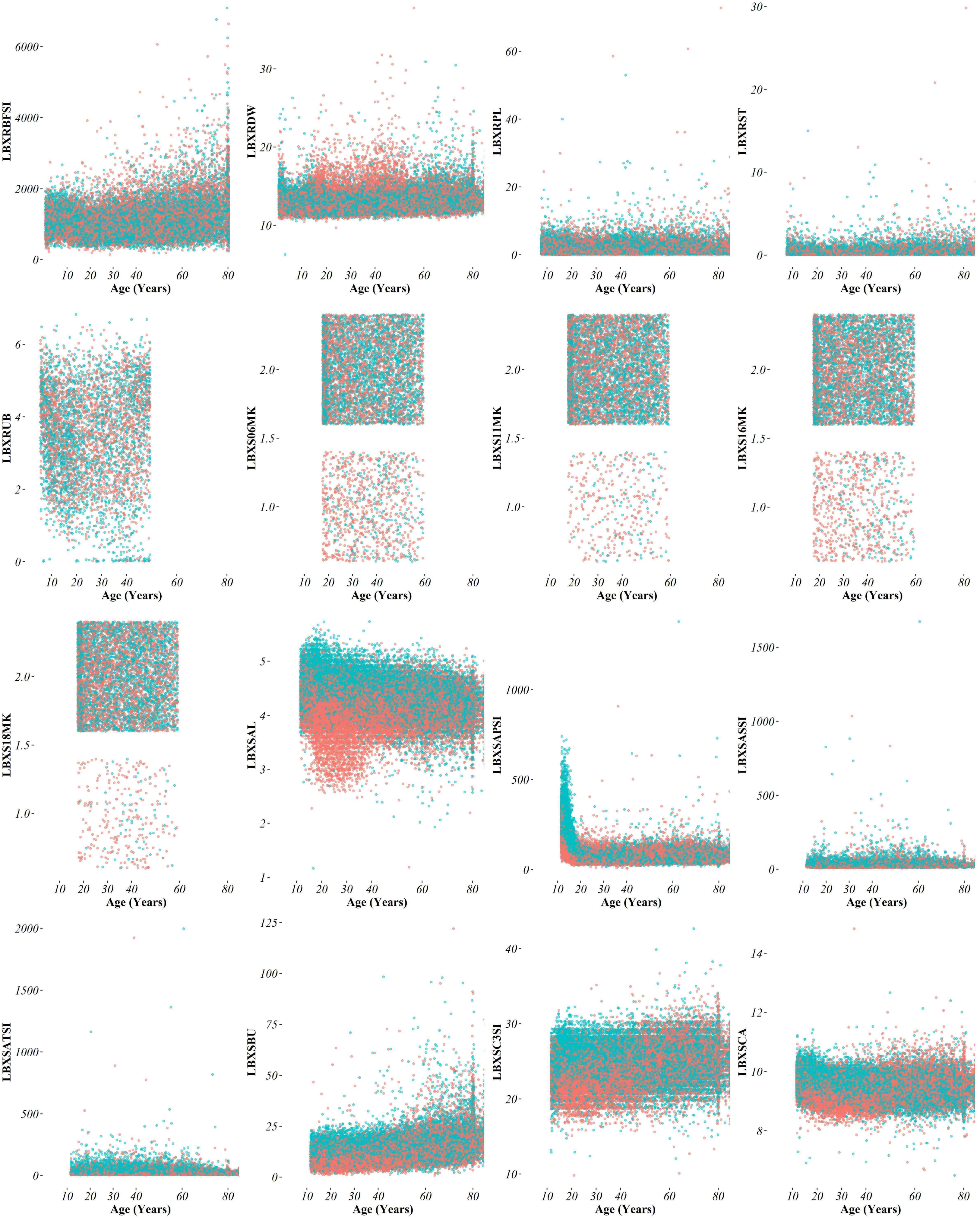

LBXRBFSI
0 2000 4000 6000
LBXRDW
10 20 30
LBXRPL
0 20 40 60
LBXRST
0 10 20 30
LBXRUB
0 2 4 6
LBXS06MK
1.0 1.5 2.0
LBXS11MK
1.0 1.5 2.0
LBXS16MK
1.0 1.5 2.0
LBXS18MK
1.0 1.5 2.0
LBXSAL
1 2 3 4 5
LBXSAPSI
0 500 1000
LBXSASSI
0 500 1000 1500
LBXSATSI
0 500 1000 1500 2000
LBXSBU
0 25 50 75 100 125
LBXSC3SI
10 20 30 40
LBXSCA
8 10 12 14
10 20 30 40 60 80
Age (Years)

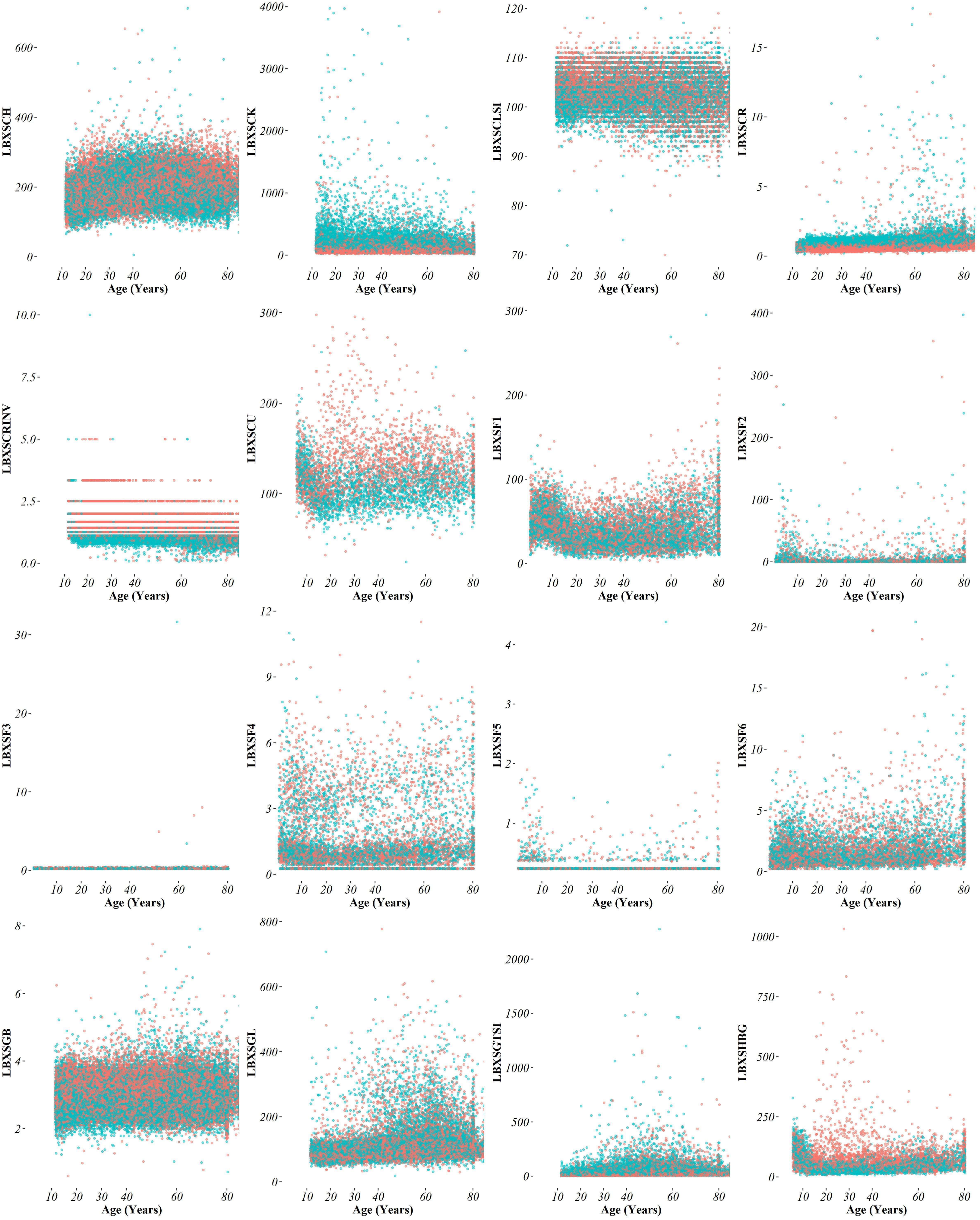

LBXSCH
Age (Years)
LBXSCK
Age (Years)
LBXSCLSI
Age (Years)
LBXSCR
Age (Years)
LBXSCRINV
Age (Years)
LBXSCU
Age (Years)
LBXSF1
Age (Years)
LBXSF2
Age (Years)
LBXSF3
Age (Years)
LBXSF4
Age (Years)
LBXSF5
Age (Years)
LBXSF6
Age (Years)
LBXSGB
Age (Years)
LBXSGL
Age (Years)
LBXSGTSI
Age (Years)
LBXSHBG
Age (Years)

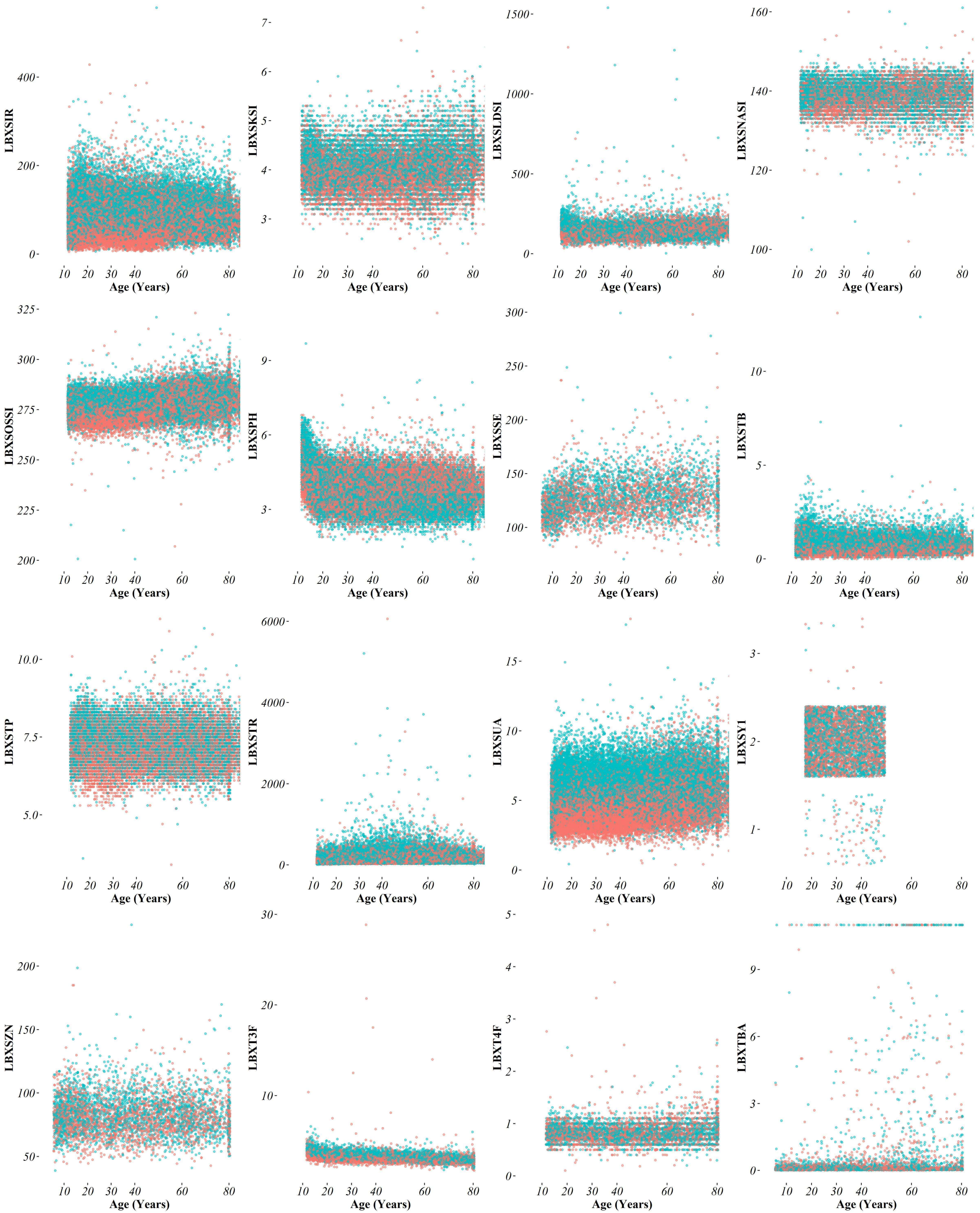
LBXSIR
LBXSKSI
LBXSLDSI
LBXSNASI
LBXSOSSI
LBXSPH
LBXSSE
LBXSTB
LBXSTP
LBXSTR
LBXSUA
LBXSY1
LBXSZN
LBXT3F
LBXT4F
LBXTBA
Age (Years)
10
20
30
40
60
80

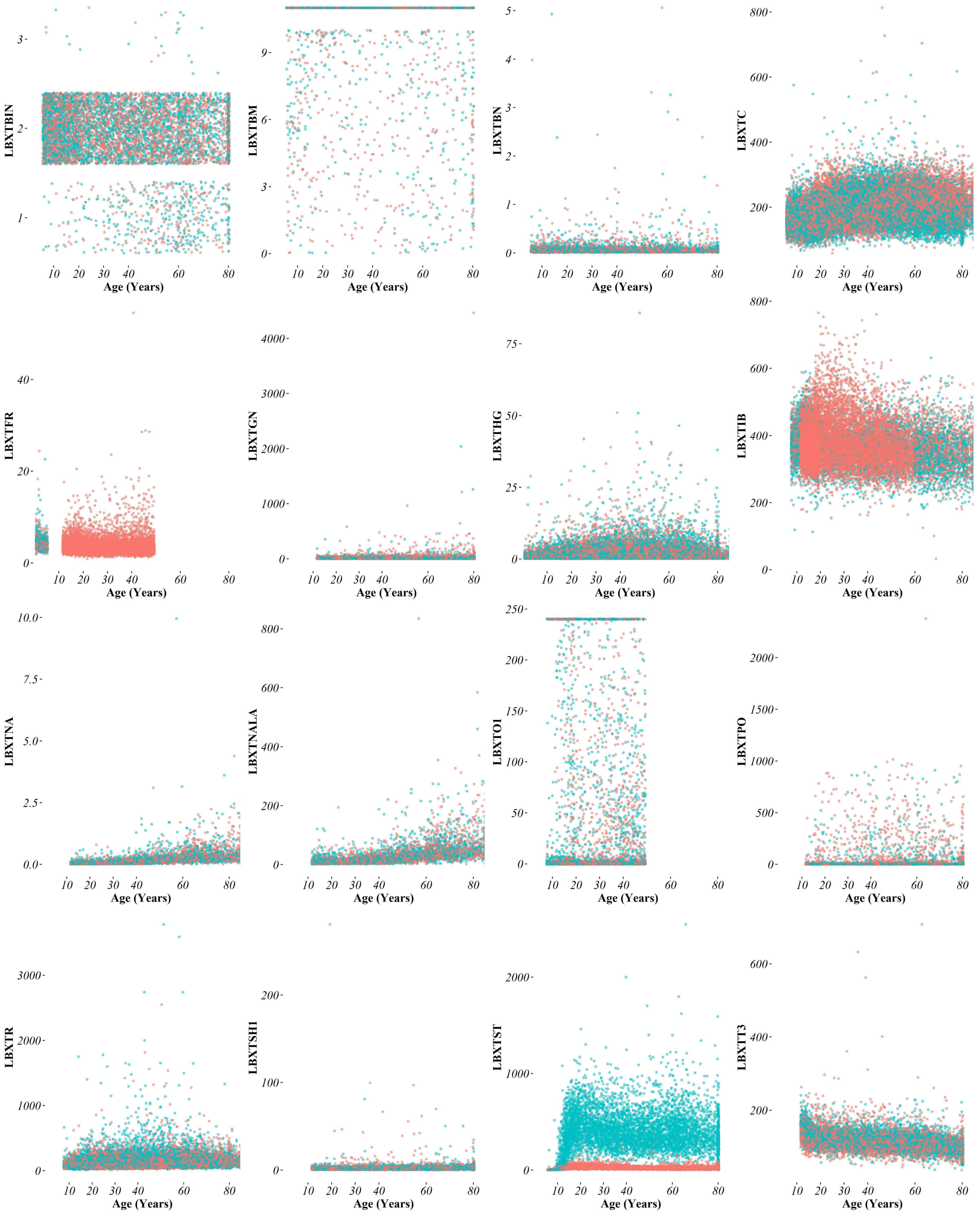

LBXTBIN
Age (Years)
LBXTBM
Age (Years)
LBXTBN
Age (Years)
LBXTC
Age (Years)
LBXTFR
Age (Years)
LBXTGN
Age (Years)
LBXTHG
Age (Years)
LBXTIB
Age (Years)
LBXTNA
Age (Years)
LBXTNALA
Age (Years)
LBXTO1
Age (Years)
LBXTPO
Age (Years)
LBXTR
Age (Years)
LBXTSH1
Age (Years)
LBXTST
Age (Years)
LBXTT3
Age (Years)

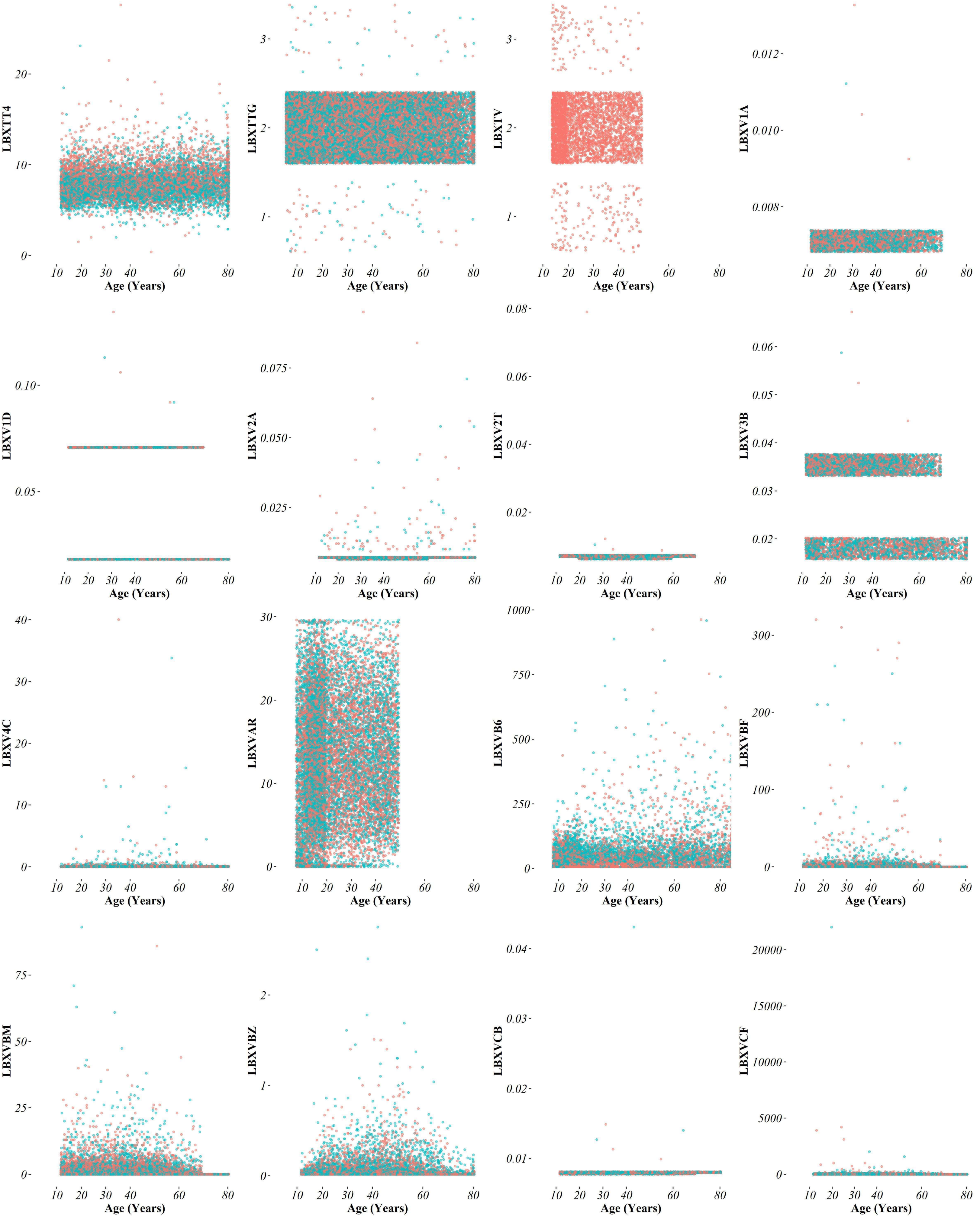
LBXTT4
LBXTTG
LBXTV
LBXV1A
LBXV1D
LBXV2A
LBXV2T
LBXV3B
LBXV4C
LBXVAR
LBXVB6
LBXVBF
LBXVBM
LBXVBZ
LBXVCB
LBXVCF
Age (Years)

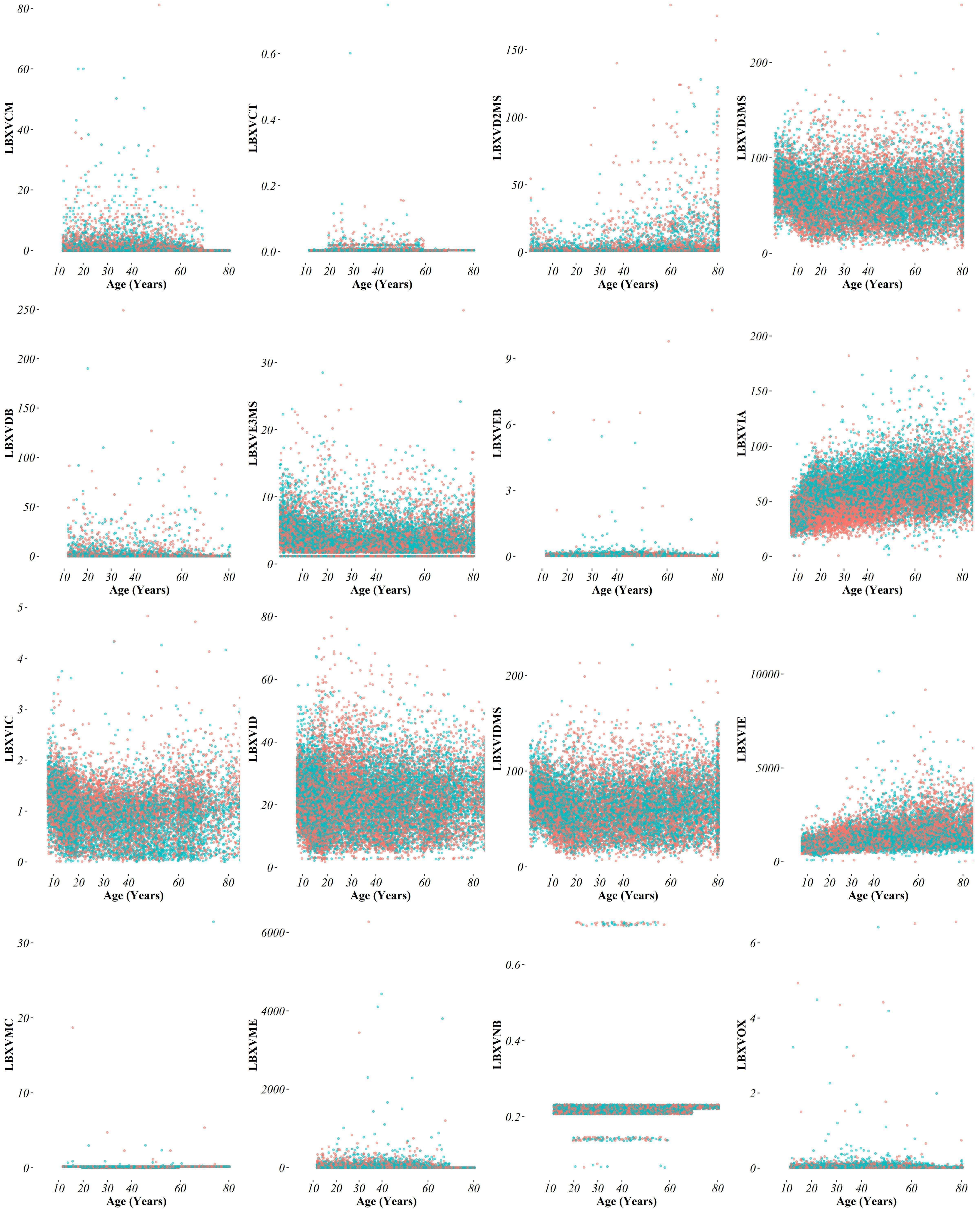
LBXVCM
Age (Years)
LBXVCT
Age (Years)
LBXVD2MS
Age (Years)
LBXVD3MS
Age (Years)
LBXVDB
Age (Years)
LBXVE3MS
Age (Years)
LBXVEB
Age (Years)
LBXVIA
Age (Years)
LBXVIC
Age (Years)
LBXVID
Age (Years)
LBXVIDMS
Age (Years)
LBXVIE
Age (Years)
LBXVMC
Age (Years)
LBXVME
Age (Years)
LBXVNB
Age (Years)
LBXVOX
Age (Years)

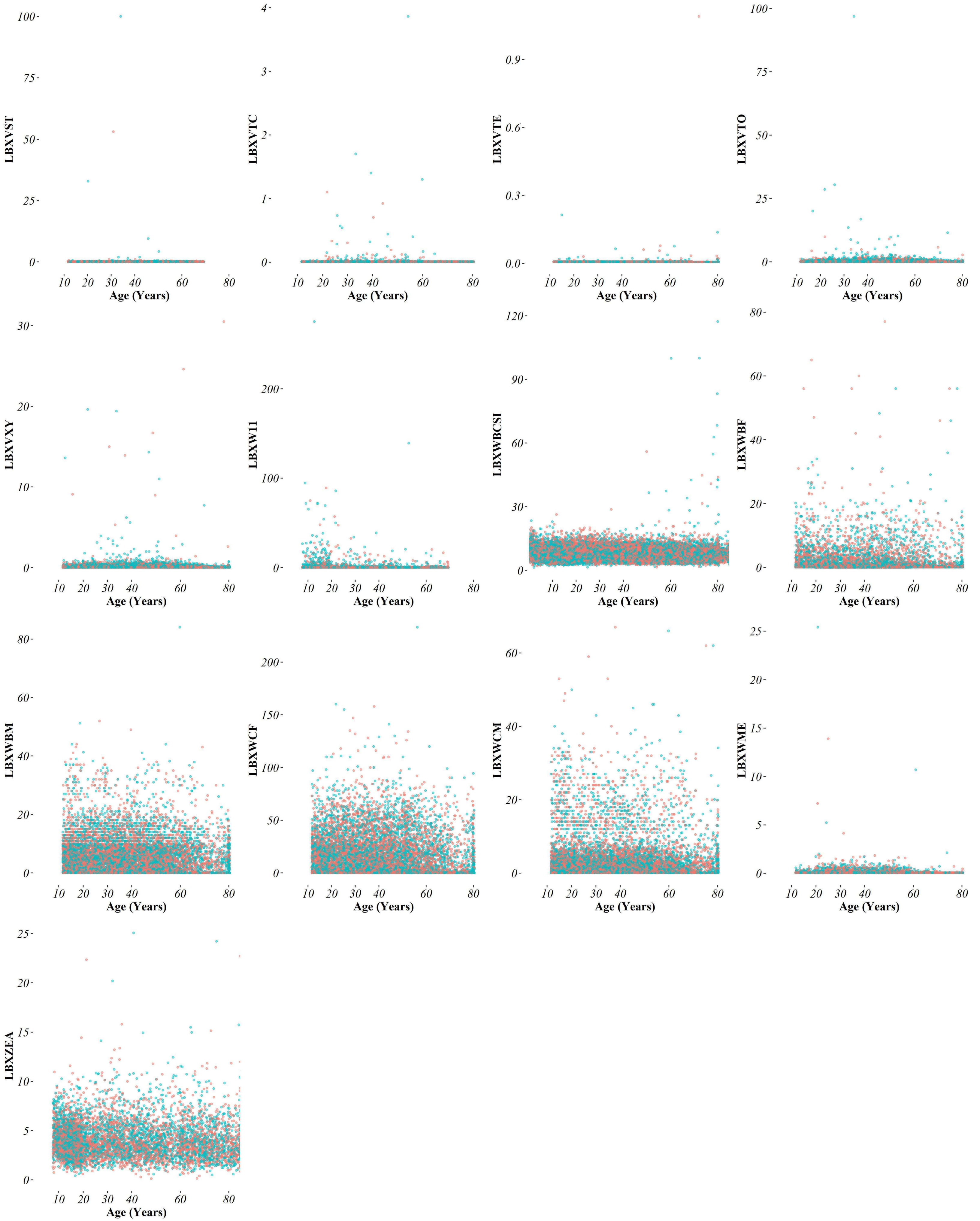
LBXVST
LBXVTC
LBXVTE
LBXVTO
LBXVXY
LBXW11
LBXWBCSI
LBXWBF
LBXWBM
LBXWCF
LBXWCM
LBXWME
LBXZEA
Age (Years)
10 20 30 40 60 80
