# Supplementary material for: Prediction of chronological and biological age from laboratory data
Source: Aging (Albany NY). 2020 May 5;12(9):7626–38. doi: 10.18632/aging.102900 (PMC7244024; doi:10.18632/aging.102900)
Supplement: Supplementary Table 1 [file aging-12-102900-s001..pdf]

Supplementary Table 1. Cumulative relative importance scores for the Top-5 and Top-10 laboratory analytes for predicting age by age group.

Group	Relative Importance
|Top-10[1,18)|	0.751
|Top-10[18,45)|	0.294
|Top-10[45,65)|	0.542
|Top-1065+|	0.225
|Top-5[1,18)|	0.567
|Top-5[18,45)|	0.169
|Top-5[45,65)|	0.464
|Top-565+|	0.144
