# Supplementary material for: Prediction of chronological and biological age from laboratory data
Source: Aging (Albany NY). 2020 May 5;12(9):7626–38. doi: 10.18632/aging.102900 (PMC7244024; doi:10.18632/aging.102900)
Supplement: Supplementary Table 2 [file aging-12-102900-s003..pdf]

Supplementary Table 2: List of 356 Laboratory Variables

Full variable names and additional information can be found here:

https://wwwn.cdc.gov/nchs/nhanes/Search/default.aspx

'LBXWBCSI',	'LBXSCRINV',	'LBXVBF',	'LBX157LA',
'LBXLYPCT',	'LBXTO1',	'LBXVBM',	'LBX167',
'LBXMOPCT',	'LBXTR',	'LBXVBZ',	'LBX167LA',
'LBXNEPCT',	'LBXTC',	'LBXVCF',	'LBX170',
'LBXEOPCT',	'LBXCRP',	'LBXVCM',	'LBX170LA',
'LBXBAPCT',	'LBXHP1',	'LBXVCT',	'LBX172',
'LBXRBCSI',	'LBXFB',	'LBXVDB',	'LBX172LA',
'LBXHGB',	'LBXBAP',	'LBXVEB',	'LBX177',
'LBXHCT',	'LBXGLU',	'LBXVME',	'LBX177LA',
'LBXMCVSI',	'LBXCPSI',	'LBXVOX',	'LBX178',
'LBXMCHSI',	'LBXIN',	'LBXVST',	'LBX178LA',
'LBXMC',	'LBXGH',	'LBXVTC',	'LBX180',
'LBXRDW',	'LBXHE1',	'LBXVTO',	'LBX180LA',
'LBXPLTSI',	'LBXHE2',	'LBXVXY',	'LBX183',
'LBXMPSI',	'LBXLA',	'LBXHA',	'LBX183LA',
'LBXME',	'LBXLACL',	'LBXHBC',	'LBX187',
'LBXVAR',	'LBXBPB',	'LBXHBS',	'LBX187LA',
'LBXT4',	'LBXBCD',	'LBX052',	'LBXD01',
'LBXTSH',	'LBXEPP',	'LBX052LA',	'LBXD01LA',
'LBXSAL',	'LBXIRN',	'LBX066',	'LBXD03',
'LBXSATSI',	'LBXTIB',	'LBX066LA',	'LBXD03LA',
'LBXSASSI',	'LBXPCT',	'LBX074',	'LBXD04',
'LBXSAPSI',	'LBXFER',	'LBX074LA',	'LBXD04LA',
'LBXSBU',	'LBXFOL',	'LBX099',	'LBXD05',
'LBXSCA',	'LBXB12',	'LBX099LA',	'LBXD05LA',
'LBXSCH',	'LBXHCY',	'LBX101',	'LBXD07',
'LBXSC3SI',	'LBXMMA',	'LBX101LA',	'LBXD07LA',
'LBXSGTSI',	'LBXTHG',	'LBX105',	'LBXF01',
'LBXSGL',	'LBXIHG',	'LBX105LA',	'LBXF01LA',
'LBXSIR',	'LBXRBF',	'LBX118',	'LBXF02',
'LBXSLDSI',	'LBXCOT',	'LBX118LA',	'LBXF02LA',
'LBXSPH',	'LBXGTC',	'LBX128',	'LBXF03',
'LBXSTB',	'LBXRPL',	'LBX128LA',	'LBXF03LA',
'LBXSTP',	'LBXRST',	'LBX138',	'LBXF04',
'LBXSTR',	'LBXVIA',	'LBX138LA',	'LBXF04LA',
'LBXSUA',	'LBXVIE',	'LBX146',	'LBXF05',
'LBXSCR',	'LBXWBF',	'LBX146LA',	'LBXF05LA',
'LBXSNASI',	'LBXWCF',	'LBX153',	'LBXF06',
'LBXSKSI',	'LBXWBM',	'LBX153LA',	'LBXF06LA',
'LBXSCLSI',	'LBXWCM',	'LBX156',	'LBXF07',
'LBXSOSSI',	'LBXWME',	'LBX156LA',	'LBXF07LA',
'LBXSGB',	'LBXV4C',	'LBX157',	'LBXF08',

'LBXF08LA',
'LBXF10',
'LBXF10LA',
'LBXPCB',
'LBXPCBLA',
'LBXTC2',
'LBXTC2LA',
'LBXTCD',
'LBXTCDLA',
'LBXGHC',
'LBXGHCLA',
'LBXHCB',
'LBXHCBLA',
'LBXHPE',
'LBXHPELA',
'LBXHXC',
'LBXHXCLA',
'LBXMIR',
'LBXMIRLA',
'LBXODT',
'LBXODTLA',
'LBXOXY',
'LBXOXYLA',
'LBXPDE',
'LBXPDELA',
'LBXPDT',
'LBXPDTLA',
'LBXTNA',
'LBXTNALA',
'LBXBHC',
'LBXBHCLA',
'LBXEPAH',
'LBXMPAH',
'LBXPFDE',
'LBXPFDO',
'LBXPFHP',
'LBXPFHS',
'LBXPFNA',
'LBXPFOA',
'LBXPFOS',
'LBXPFSA',
'LBXPFUA',
'LBXVID',
'LBXALC',
'LBXBEC',
'LBXCBC',
'LBXCRY',
'LBXLUZ',
'LBXLYC',
'LBXP1',
'LBXP2',
'LBX087',
'LBX087LA',
'LBX110',
'LBX110LA',
'LBX149',
'LBX149LA',
'LBX151',
'LBX151LA',
'LBX189',
'LBX189LA',
'LBX194',
'LBX194LA',
'LBX195',
'LBX195LA',
'LBX196',
'LBX196LA',
'LBX206',
'LBX206LA',
'LBXDIE',
'LBXDIELA',
'LBXALD',
'LBXALDLA',
'LBXEND',
'LBXENDLA',
'LBXMS1',
'LBXM1',
'LBXSY1',
'LBXV1A',
'LBXV1D',
'LBXV1E',
'LBXV2A',
'LBXV2C',
'LBXV2T',
'LBXV3B',
'LBXVCB',
'LBXVDM',
'LBXVDP',
'LBXVHE',
'LBXVMC',
'LBX2DF',
'LBXV2P',
'LBXVNB',
'LBXTFR',
'LBXVIC',
'LBXPT21',
'LBXHDD',
'LBXPFBS',
'LBXVB6',
'LBXATC',
'LBXACY',
'LBXBCC',
'LBXCLC',
'LBXCLZ',
'LBXDTC',
'LBXLCC',
'LBXLUT',
'LBXPHF',
'LBXPHE',
'LBXZEA',
'LBXACR',
'LBXGLY',
'LBXS06MK',
'LBXS11MK',
'LBXS16MK',
'LBXS18MK',
'LBXIGE',
'LBXID2',
'LBXID1',
'LBXIE1',
'LBXIE5',
'LBXII6',
'LBXIM6',
'LBXF13',
'LBXIF1',
'LBXIF2',
'LBXIW1',
'LBXIG5',
'LBXIG2',
'LBXIT7',
'LBXIT3',
'LBXF24',
'LBXIM3',
'LBXW11',
'LBXE72',
'LBXE74',
'LBXGLT',
'LBXAPB',
'LBXVTE',
'LBXDWT',
'LBXDWS',
'LBX4PA',
'LBXPLP',
'LBXRBFSI',
'LBXFOLSI',
'LBXHCR',
'LBXH2RL',
'LBXATG',
'LBXT3F',
'LBXT4F',
'LBXTGN',
'LBXTSH1',
'LBXTPO',
'LBXTT3',
'LBXTT4',
'LBXSF1',
'LBXSF2',
'LBXVIDMS',
'LBXVD2MS',
'LBXVD3MS',
'LBXVE3MS',
'LBX06',
'LBX11',
'LBX16',
'LBX18',
'LBXMEA',
'LBXMUM',
'LBXRUB',
'LBXTTG',
'LBXSCK',
'LBXSCU',
'LBXSSE',
'LBXSZN',
'LBXSF3',
'LBXSF4',
'LBXSF5',
'LBXSF6',
'LBXBGE',
'LBXBGM',
'LBXBSE',
'LBXBMN',
'LBXTBA',
'LBXTBN',
'LBXTBIN',
'LBXTBM',
'LBXTST',
'LBXEST',
'LBXSHBG'
