# Supplementary material for: Prediction of chronological and biological age from laboratory data
Source: Aging (Albany NY). 2020 May 5;12(9):7626–38. doi: 10.18632/aging.102900 (PMC7244024; doi:10.18632/aging.102900)
Supplement: Supplementary Table 3 [file aging-12-102900-s002..pdf]

Supplementary Table 3

Number of missing values for each variable

| **Laboratory Variable** | **Number Missing** |
|---|---|
| LBXV2P | 63747 |
| LBXLA | 63601 |
| LBXLACL | 63601 |
| LBXEND | 63514 |
| LBXENDLA | 63514 |
| LBX195 | 63479 |
| LBX195LA | 63479 |
| LBX206 | 63455 |
| LBX206LA | 63455 |
| LBXVDP | 63442 |
| LBXV2C | 63427 |
| LBXVDM | 63418 |
| LBXDIE | 63415 |
| LBXDIELA | 63415 |
| LBX194 | 63414 |
| LBX194LA | 63414 |
| LBX189 | 63413 |
| LBX189LA | 63413 |
| LBXVHE | 63411 |
| LBXV1E | 63400 |
| LBXTSH | 63386 |
| LBXT4 | 63382 |
| LBXV1A | 63375 |
| LBXV2T | 63375 |
| LBX151 | 63351 |
| LBX151LA | 63351 |
| LBX196 | 63351 |
| LBX196LA | 63351 |
| LBX110 | 63348 |
| LBX110LA | 63348 |
| LBX149 | 63348 |
| LBX149LA | 63348 |
| LBX087 | 63338 |
| LBX087LA | 63338 |
| LBXALD | 63305 |
| LBXALDLA | 63305 |
| LBXM1 | 63166 |
| LBXF08 | 62952 |
| LBXF08LA | 62952 |
| LBXF10 | 62819 |
| LBXF10LA | 62819 |

| | |
|---|---|
| LBXD07 | 62817 |
| LBXD07LA | 62817 |
| LBXTC2 | 62798 |
| LBXTC2LA | 62798 |
| LBXF06 | 62792 |
| LBXF06LA | 62792 |
| LBXF04 | 62776 |
| LBXF04LA | 62776 |
| LBXF07 | 62776 |
| LBXF07LA | 62776 |
| LBXPCB | 62776 |
| LBXPCBLA | 62776 |
| LBXHXC | 62775 |
| LBXHXCLA | 62775 |
| LBXD04 | 62774 |
| LBXD04LA | 62774 |
| LBXD05 | 62770 |
| LBXD05LA | 62770 |
| LBXD03 | 62761 |
| LBXD03LA | 62761 |
| LBXF01 | 62761 |
| LBXF01LA | 62761 |
| LBXF03 | 62761 |
| LBXF03LA | 62761 |
| LBXF05 | 62754 |
| LBXF05LA | 62754 |
| LBXTCD | 62754 |
| LBXTCDLA | 62754 |
| LBXDWS | 62743 |
| LBXDWT | 62739 |
| LBXF02 | 62735 |
| LBXF02LA | 62735 |
| LBXD01 | 62727 |
| LBXD01LA | 62727 |
| LBXSSE | 62710 |
| LBXSZN | 62710 |
| LBXSCU | 62709 |
| LBXMEA | 62494 |
| LBXMUM | 62494 |
| LBXRUB | 62494 |
| LBX052 | 62413 |
| LBX052LA | 62413 |
| LBXVST | 62359 |
| LBXH2RL | 62166 |

| | |
|---|---|
| LBXHPE | 61935 |
| LBXHPELA | 61935 |
| LBXOXY | 61868 |
| LBXOXYLA | 61868 |
| LBXODT | 61864 |
| LBXODTLA | 61864 |
| LBXPDT | 61813 |
| LBXPDTLA | 61813 |
| LBXFB | 61786 |
| LBXHCB | 61786 |
| LBXHCBLA | 61786 |
| LBX172 | 61776 |
| LBX172LA | 61776 |
| LBXSY1 | 61775 |
| LBX170 | 61768 |
| LBX170LA | 61768 |
| LBXVTE | 61754 |
| LBXHP1 | 61750 |
| LBXPCT | 61748 |
| LBXGHC | 61717 |
| LBXGHCLA | 61717 |
| LBX177 | 61712 |
| LBX177LA | 61712 |
| LBX157 | 61704 |
| LBX157LA | 61704 |
| LBXMIR | 61698 |
| LBXMIRLA | 61698 |
| LBX099 | 61696 |
| LBX099LA | 61696 |
| LBX167 | 61687 |
| LBX167LA | 61687 |
| LBX066 | 61682 |
| LBX066LA | 61682 |
| LBX156 | 61673 |
| LBX156LA | 61673 |
| LBX128 | 61657 |
| LBX128LA | 61657 |
| LBX105 | 61655 |
| LBX105LA | 61655 |
| LBX146 | 61643 |
| LBX146LA | 61643 |
| LBX178 | 61642 |
| LBX178LA | 61642 |
| LBX138 | 61640 |

| | |
|---|---|
| LBX138LA | 61640 |
| LBX183 | 61639 |
| LBX183LA | 61639 |
| LBX118 | 61637 |
| LBX118LA | 61637 |
| LBX180 | 61636 |
| LBX180LA | 61636 |
| LBX187 | 61633 |
| LBX187LA | 61633 |
| LBX074 | 61631 |
| LBX074LA | 61631 |
| LBX153 | 61631 |
| LBX153LA | 61631 |
| LBX101 | 61627 |
| LBX101LA | 61627 |
| LBXBHC | 61611 |
| LBXBHCLA | 61611 |
| LBXTNA | 61580 |
| LBXTNALA | 61580 |
| LBXPDE | 61547 |
| LBXPDELA | 61547 |
| LBXIF2 | 61300 |
| LBXE74 | 61268 |
| LBXE72 | 61248 |
| LBXW11 | 61236 |
| LBXF24 | 61233 |
| LBXIM3 | 61233 |
| LBXIF1 | 61231 |
| LBXF13 | 61230 |
| LBXIT3 | 61229 |
| LBXIT7 | 61228 |
| LBXIM6 | 61226 |
| LBXIW1 | 61226 |
| LBXIG5 | 61226 |
| LBXIG2 | 61226 |
| LBXII6 | 61220 |
| LBXIGE | 61216 |
| LBXID2 | 61215 |
| LBXIE5 | 61215 |
| LBXID1 | 61214 |
| LBXIE1 | 61214 |
| LBXACR | 61025 |
| LBXGLY | 60918 |
| LBXVMC | 60835 |

| | |
|---|---|
| LBXS06MK | 60737 |
| LBXS11MK | 60737 |
| LBXS16MK | 60737 |
| LBXS18MK | 60737 |
| LBXVNB | 60624 |
| LBXSHBG | 60604 |
| LBXVB6 | 60552 |
| LBXATC | 60516 |
| LBXACY | 60516 |
| LBXBCC | 60516 |
| LBXCLC | 60516 |
| LBXCLZ | 60516 |
| LBXDTC | 60516 |
| LBXLCC | 60516 |
| LBXLUT | 60516 |
| LBXPHF | 60516 |
| LBXPHE | 60516 |
| LBXZEA | 60516 |
| LBXV1D | 60488 |
| LBXP1 | 60483 |
| LBXP2 | 60483 |
| LBX2DF | 60468 |
| LBXV2A | 60450 |
| LBXHDD | 60439 |
| LBXTBIN | 60420 |
| LBXTBM | 60419 |
| LBXVCB | 60416 |
| LBXTBA | 60416 |
| LBXTBN | 60415 |
| LBXEST | 60413 |
| LBXV3B | 60407 |
| LBXSF4 | 60061 |
| LBXSF3 | 60056 |
| LBXSF5 | 60056 |
| LBXSF6 | 60053 |
| LBXHCR | 60019 |
| LBX06 | 59880 |
| LBX11 | 59880 |
| LBX16 | 59880 |
| LBX18 | 59880 |
| LBXVME | 59688 |
| LBXVCF | 59569 |
| LBXVBF | 59402 |
| LBXVCT | 59251 |

| | |
|---|---|
| LBXVDB | 59237 |
| LBXVBM | 59221 |
| LBXVCM | 59213 |
| LBXVBZ | 59212 |
| LBXVEB | 59187 |
| LBXV4C | 59097 |
| LBXVTC | 59059 |
| LBXVTO | 59028 |
| LBXWME | 59008 |
| LBXVXY | 58974 |
| LBXWCF | 58971 |
| LBXWBM | 58921 |
| LBXWBF | 58920 |
| LBXVOX | 58912 |
| LBXWCM | 58900 |
| LBXCPSI | 58345 |
| LBXTFR | 57662 |
| LBXSF2 | 57349 |
| LBXSF1 | 57330 |
| LBXPFBS | 57324 |
| LBXTPO | 57007 |
| LBXATG | 56981 |
| LBXTT4 | 56962 |
| LBXT3F | 56927 |
| LBXTGN | 56923 |
| LBXTT3 | 56920 |
| LBXT4F | 56915 |
| LBXTSH1 | 56915 |
| LBXEPAH | 55924 |
| LBXPFHP | 55924 |
| LBXPFOA | 55924 |
| LBXPFOS | 55924 |
| LBXPFSA | 55924 |
| LBXAPB | 55334 |
| LBXVIC | 54232 |
| LBXPT21 | 54131 |
| LBXGLT | 53957 |
| LBXMPAH | 53931 |
| LBXPFDE | 53931 |
| LBXPFDO | 53931 |
| LBXPFHS | 53931 |
| LBXPFNA | 53931 |
| LBXPFUA | 53931 |
| LBXTST | 53494 |

| | |
|---|---|
| LBXTO1 | 53475 |
| LBXME | 53403 |
| LBXVAR | 53403 |
| LBXMS1 | 52709 |
| LBXVE3MS | 52034 |
| LBXVIDMS | 51856 |
| LBXVD2MS | 51855 |
| LBXVD3MS | 51855 |
| LBXBGE | 51124 |
| LBXBGM | 51122 |
| LBXBSE | 50995 |
| LBXBMN | 50995 |
| LBXEPP | 49337 |
| LBXBAP | 49238 |
| LBXTIB | 48966 |
| LBXIRN | 48904 |
| LBXSCK | 48772 |
| LBXCBC | 47149 |
| LBXMMA | 47045 |
| LBXCRY | 46691 |
| LBXALC | 46609 |
| LBXLYC | 46608 |
| LBXLUZ | 46607 |
| LBXBEC | 46600 |
| LBXVID | 46485 |
| LBXTTG | 45929 |
| LBXTR | 45441 |
| LBX4PA | 44553 |
| LBXPLP | 44552 |
| LBXFER | 44067 |
| LBXRST | 43867 |
| LBXIN | 43375 |
| LBXFOLSI | 43101 |
| LBXHCY | 43023 |
| LBXSCRINV | 42926 |
| LBXRBFSI | 42586 |
| LBXRPL | 42564 |
| LBXGTC | 41678 |
| LBXHE2 | 41512 |
| LBXVIE | 40849 |
| LBXVIA | 40806 |
| LBXFOL | 40592 |
| LBXRBF | 40395 |
| LBXGLU | 39766 |

| | |
|---|---|
| LBXB12 | 35759 |
| LBXHE1 | 34989 |
| LBXCRP | 25370 |
| LBXTHG | 25161 |
| LBXCOT | 17327 |
| LBXIHG | 17298 |
| LBXMC | 15143 |
| LBXSLDSI | 10903 |
| LBXSASSI | 10901 |
| LBXSATSI | 10896 |
| LBXSC3SI | 10884 |
| LBXSGB | 10883 |
| LBXSTP | 10882 |
| LBXSIR | 10876 |
| LBXSCA | 10867 |
| LBXSTB | 10853 |
| LBXSTR | 10849 |
| LBXSUA | 10834 |
| LBXSCLSI | 10833 |
| LBXSGTSI | 10830 |
| LBXSPH | 10830 |
| LBXSKSI | 10830 |
| LBXSAPSI | 10829 |
| LBXSCH | 10829 |
| LBXSOSSI | 10829 |
| LBXSBU | 10826 |
| LBXSNASI | 10826 |
| LBXSCR | 10824 |
| LBXSAL | 10822 |
| LBXSGL | 10821 |
| LBXGH | 10087 |
| LBXHA | 7464 |
| LBXLYPCT | 7179 |
| LBXMOPCT | 7179 |
| LBXNEPCT | 7179 |
| LBXEOPCT | 7179 |
| LBXBAPCT | 7179 |
| LBXWBCSI | 7031 |
| LBXPLTSI | 7030 |
| LBXMPSI | 7030 |
| LBXRBCSI | 7029 |
| LBXHGB | 7029 |
| LBXHCT | 7029 |
| LBXMCVSI | 7029 |

| | |
|---|---|
| LBXMCHSI | 7029 |
| LBXRDW | 7029 |
| LBXBPB | 6594 |
| LBXBCD | 6594 |
| LBXTC | 4240 |
| LBXHBC | 4083 |
| LBXHBS | 1887 |
